# Surface Mapping of Functionalized Two-Dimensional Nanosheets: Graphene Oxide and MXene Materials

**DOI:** 10.1021/acs.langmuir.4c05106

**Published:** 2025-05-12

**Authors:** Madeline L. Buxton, Justin Brackenridge, Valeriia Poliukhova, Dhriti Nepal, Timothy J. Bunning, Vladimir V. Tsukruk

**Affiliations:** † School of Materials Science and Engineering, 1372 Georgia Institute of Technology, Atlanta, Georgia 30332, United States; ‡ Air Force Research Lab, Materials and Manufacturing Directorate, 2941 Hobson Way, Wright-Patterson AFB, Ohio 45433, United States

## Abstract

In this study, we characterized the morphology, composition, and surface properties of individual flakes of graphene oxide and Ti_3_C_2_T_*x*_ MXene chemically modified with ethylenediamine, dopamine, and (3-aminopropyl) triethoxysilane (APTES). Individual monolayers of modified Ti_3_C_2_T_*x*_ MXene and graphene oxide nanosheets were deposited using the Langmuir–Blodgett technique. We compared the chemical surface modification of these two-dimensional (2D) flakes by employing advanced atomic force microscopy (AFM) modes, including quantitative nanomechanical (QNM) mode, Kelvin–Probe force microscopy (KPFM), and Nano-IR imaging. This approach reveals the distribution of mechanical, electrical, and chemical properties on individual flakes at the nanoscale. QNM analysis confirms that the flakes exhibited full surface coverage after the chemical modification process. In modified MXene flakes, we observed a decrease in apparent elastic modulus and an increase in adhesion of up to four times after their functionalization. Nano-IR imaging demonstrates that chemical modification uniformity is highest for graphene oxide species, while the complex surface distribution was observed for dopamine-modified MXene flakes, with a difference between the inner flat surface and their edges. KPFM indicates greater uniformity of surface electrical potential in differently modified graphene oxide, while a significant increase in surface potential of MXene flakes is seen when modified with dopamine. We suggest that a combination of the added dielectric layer and different grafting densities across the flakes is responsible for the increased or changes in apparent surface potential. Overall, a combination of AFM probing modes is needed for understanding how these functionalized nanosheets can be integrated into diverse polymer matrices.

## Graphical abstract

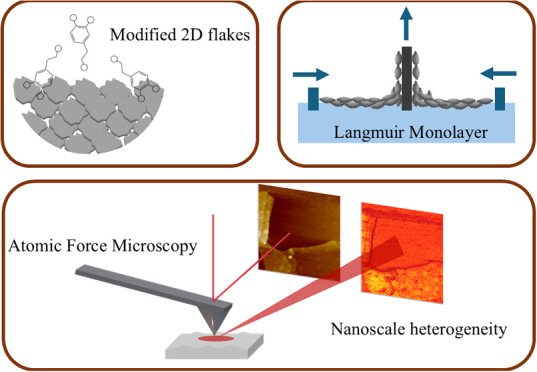

## Introduction

The use of functional two-dimensional (2D) materials in diverse polymer composites requires new and improved surface functionalities to take full advantage of their high specific surface area, high Young’s modulus, tailored interfacial adhesion, and good thermal conductivity.^1,2^ These materials are not only of interest for heterostructure electronic materials but also provide critical improvements and additional functionalities when utilized as coatings, binders, and nanofillers. Two prominent 2D nanomaterials considered for such applications are MXene and graphene derivatives. Graphene oxide and other graphene derivatives appeal due to their versatility, scalability, and low cost.^1,3^ MXene competes with graphene oxide, as they share similar chemical and physical properties, including high energy storage capabilities, large surface area, significantly high concentration of negative surface charges, and hydrophilicity.^2,4^ Moreover, MXene exceeds graphene oxide in terms of strength, modulus, and conductivity, while maintaining comparable dispersibility in water and polar solvents.^4^ There are over 40 different known compositions of MXene composed of a transition metal and carbon and/or nitrogen.^5^ Ti_3_C_2_T_*x*_ is the most common and environmentally stable composition with abundant surface functional groups; hence, it is used here.^6^ Due to their interlayer stability, Ti_3_C_2_T_*x*_ can maintain its properties, specifically conductivity, which is usually the first indication of degradation. However, stability under proper storage has been shown over multiple years.^7^

Different functionalization methods have been proposed for enhancing the stability of MXene and graphene flakes for improving their integration into various polymer matrices.^8,9^ Surface chemistry modifications can provide increased tunability of properties and better control over interfacial interactions in heterostructured nanocomposite materials.^10^ A prominent example of 2D heterostructure materials’ potential lies in interlayer electron or ion transfer, which can be improved by precise molecular organization and the native ion binding properties. These properties often depend on the specific surface modifications applied to the system.^11^ To date, different entities, such as amines,^12^ quinones,^13^ and catechol structures,^14^ have been utilized to modify 2D nanosheets due to the abundance of hydroxyl terminations, allowing for high surface affinity for diverse covalent bonding.^15^

Several popular chemical species are widely used for current 2D material modifications. For instance, dopamine has been used extensively for graphene oxide flakes because of its strong adherence to a variety of surfaces, including carbon materials and polymer resins.^16,17^ Dopamine modification is often employed as an adhesive to enhance charge transfer and increase interlaminar spacing via covalent bonding to MXene surfaces.^18^ It can improve the mechanical integration of flakes into host matrices by reducing π–π interstacking,^19^ and it offers a less toxic alternative for graphene oxide-epoxy composites compared to traditional reducing agents like hydrazine that can significantly thin original flakes and change their surface chemistry.^17,20^

Ethylenediamine (EDA) has been shown to induce cross-linking between graphene oxide and MXene by forming covalent bonding with graphene oxide flakes and noncovalent bonding with MXene surfaces through van der Waals forces and π–π stacking interactions.^21^ The induced cross-linking between disparate flakes within a system is instigated due to the presence of two amine groups that ultimately improve mechanical strength and interlayers.^21^

Amines have been proposed to modify graphene oxide and MXene via covalent bonding to maintain a laminated structure and stabilize the mechanical properties of large-area graphene paper.^22^ Amine groups show strong interlayer bonding for both materials, contributing to increased interfacial adhesion, and can be completed under mild conditions, which is ideal for solution processing.^23,24^ Furthermore, these modifications protect vulnerable MXene flakes from oxidative impacts by forming a protective coating along the flakes.^24^ Finally, (3-aminopropyl) triethoxysilane (APTES) has been shown to covalently bond with MXene by covalently bonding to the MXene surface via the formation of Si–O bonds.^25^ This bonding provides further surface protection, postfunctionalization ability, improved stability, and adjusting the hydrophilicity of surfaces.^26^

This work aims to investigate different ways for understanding surface functionalization of popular 2D materials with a focus on the distribution of localized surface properties as influenced by heterogeneous surfaces and adsorption, grafting, and growth of organic molecules. Localized functionalization discrepancies as well as their resulting property changes on the nanoscale remain unclear, as most of the research has focused on bulk chemical and structural characterization.^9,12,16^ While chemical bonding routines have been well established as discussed above, this study addresses the way in which these molecules conform to individual flakes via comparison between graphene oxide and MXene flakes. Spatial functionality distribution needs further exploration to better understand these functionalization methods and their implications for diverse interfacial engineering.^14,18^

Here, the Langmuir–Blodgett (LB) technique was utilized to fabricate monolayer films on atomically flat substrates, enabling advanced AFM probing modes to be applied to individual monolayer flakes (Figure 1). The chemical surface modifications of these flakes were investigated using advanced AFM modes that go beyond conventional high-resolution AFM topography measurements. These complementary techniques include quantitative nanomechanical (QNM) mode, Kelvin–Probe force microscopy (KPFM), and Nano-IR imaging, which probe the mechanical, electrical, and chemical properties of the surfaces of the modified individual flakes and surrounding surface areas.^11,27,28^ Although recent research has mainly concentrated on topographical characterization,^29,30^ leveraging a combination of complementary AFM modes can unlock deeper insights into the diverse surface properties of these materials as candidates for integration into multifunctional nanocomposites.

**Figure 1:**
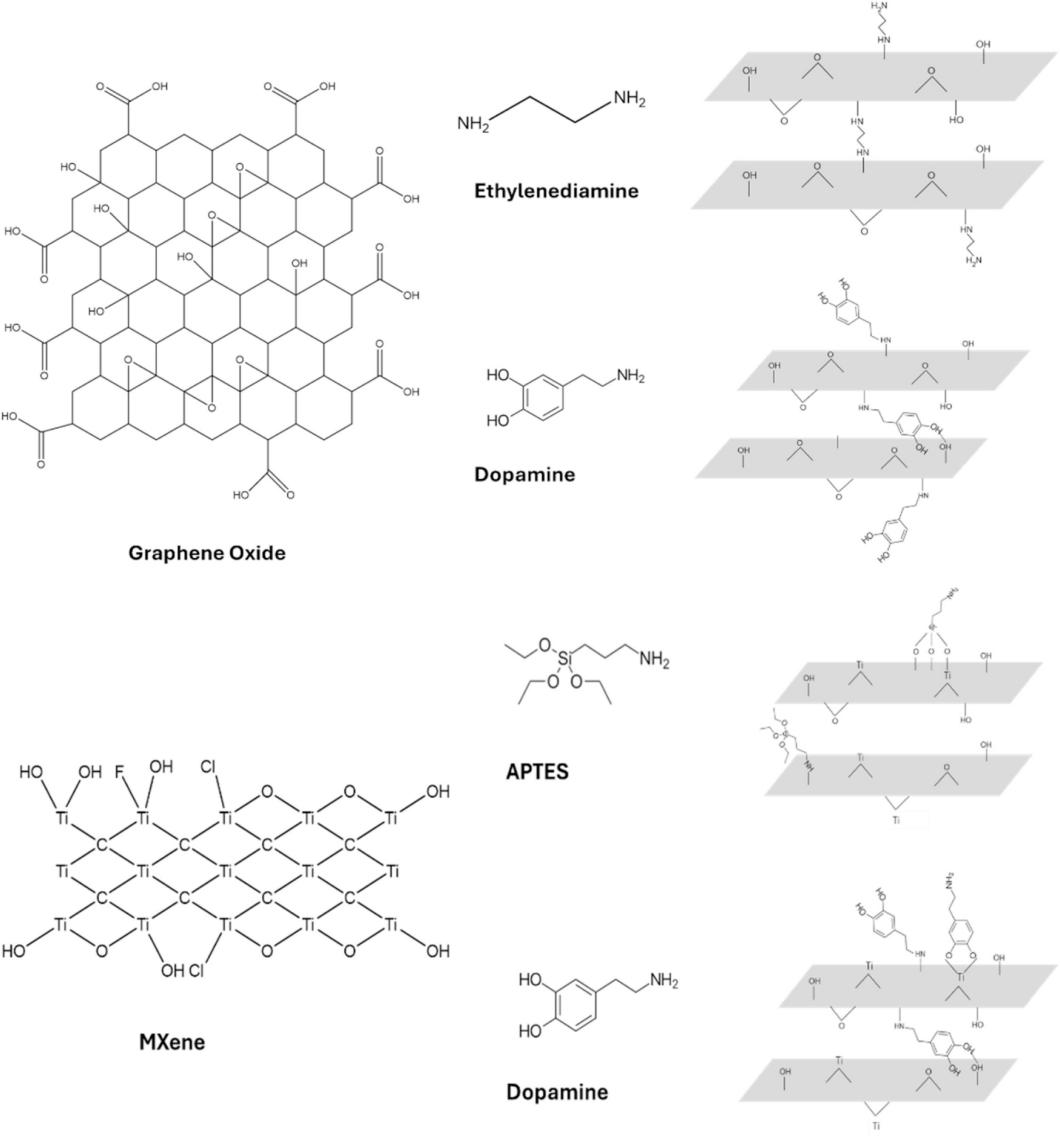
Chemical schematics of graphene oxide (top) and MXene (bottom) flakes showing surface functional groups and their bonding abilities with EDA and dopamine, and APTES, respectively.

## Experimental: Materials and Methods

### Choice of Materials

As is known, graphene oxide and MXene exhibit diverse bonding sites and can be functionalized through accessible chemical reactions (Figure 1).^14,31^

The selected molecules for functionalization were chosen because their bonding mechanisms have already been studied and shown to effectively adhere to the surface of MXene and graphene oxide flakes, as discussed above.

Amines were specifically chosen due to prior research indicating strong adhesion between MXene flakes and the surfaces of amine-modified carbon fibers.^32^ In fact, amine-modified carbon fibers demonstrate stronger bonding with MXene compared to unmodified carbon fibers.^15^ Therefore, functionalizing graphene oxide with amine molecules should enhance bonding to MXene, especially in heterostacks of alternating 2D flakes. Similarly, dopamine has been utilized with both MXene and graphene oxide, with traditional chemical characterization methods confirming the bonding mechanisms by which dopamine interacts with the surface functional groups of the flakes.^12,36^ APTES is employed because of additional postfunctionalization opportunities, improving the oxygen stability of MXene and facilitating hydrophilic adjustments.^26^ APTES can bond differently to the surface of the MXene, as the organosilyl group interacts with the titanium present in MXene (Figure 1). Three surface modifications were chosen for this study, with some limitations imposed by their surface reactivity. To this end, target amine groups have been proven to bind to both MXene and graphene oxide covalently as well as interlayer interactions as discussed previously. Fundamentally, directly comparing the way in which dopamine binds to each species was chosen due to its indicated strong bonding.

### Ti_3_C_2_T_*x*_ MXene Synthesis

In this study, we used delaminated Ti_3_C_2_T_*x*_ MXene flakes supplied by the Nanomaterials Group at Drexel University.^33^ The Ti_3_C_2_T_*x*_ was synthesized through a selective wet-chemical etching process that removed aluminum from the Ti_3_AlC_2_ MAX phase from Carbon Ukraine, Ltd. Initially, the Ti_3_AlC_2_ MAX phase was purified by washing with hydrochloric acid (HCl) to eliminate impurities. It was then immersed in 20 mL of an etchant solution composed of hydrofluoric acid (HF), HCl, and water in a volume ratio of 1:6:3 and stirred at 35 °C for 24 h. The etchant contained HF (48–51 wt %, Acros Organics) and HCl (37 wt %, Fisher Scientific).

For the delamination step, 1 g of lithium chloride (LiCl, 99%, Alfa Aesar) per gram of etched Ti_3_AlC_2_ MAX was dissolved in 50 mL of deionized water and stirred at 300 rpm at room temperature for 24 h to produce delaminated Ti_3_C_2_T_*x*_ MXenes. The resulting material was washed with deionized water and centrifuged at 3500 rpm for 5 min. After the supernatant was discarded, the MXenes were redispersed by manual shaking, and the washing cycles were repeated until the pH reached 6. Finally, the solution was centrifuged at 3500 rpm for 60 min to collect MXene flakes ranging from single layer to few-layer thickness.

MXene flakes were extracted from a concentrated sediment containing delaminated MXene, redispersed in ultrapure deionized water, hand-shaken, and centrifuged for 1 h at 3500 rpm. The initial colloidal solution contained 0.0042 wt % MXene after preparation. Two separate MXene solutions were prepared by centrifuging 5 μL of 0.0042 wt % MXene solution at 3500 rpm and extracting the supernatant until 0.5 wt % concentration of MXene in water was reached. All specimens were stored at −80C prior to use to maintain stability and prevent oxidation.

### Graphene Oxide Preparation

Graphene oxide was prepared from 325 mesh graphite following the well-known Hummers method.^34,35^ Initial wt % after graphene oxide preparation was estimated at 0.63 wt %. The solution was diluted to 0.5 wt % by adding ultrapure deionized water and sonicating for 10 min to disperse.

### Chemical Modification of 2D Flakes

Modification of MXene with dopamine was accomplished via a solution-based method as previously established.^18^ MXene at a concentration of 1 mg/mL was mixed with 1 mg/mL dopamine hydrochloride, both dispersed in DI water, at room temperature, and stirred for 24 h. The resulting suspension was rinsed via centrifugation to remove excess dopamine three times and resuspended in DI water. The resulting mixture was used at a concentration of 0.05 mg/mL.

MXene modification with APTES was similarly mixed in solution. 1 mg/mL MXene concentration was mixed in a 1:3 ratio of water and ethanol. 1 mg/mL of APTES was added to the solution and stirred at room temperature for 24 h. After stirring, the solution was rinsed via centrifugation three times to remove excess APTES and resuspended in DI water. The resulting mixture was used at a concentration of 0.05 mg/mL.

Graphene oxide was modified with dopamine by mixing 1 mg/mL graphene oxide and 1 mg/mL dopamine hydrochloride in a pH 8 dispersion of DI water and Tris-HCl buffer. The mixture was washed via centrifugation three times and redispersed in DI water, resulting in a neutral pH and filtering out unbonded dopamine (DOPA).^12^

Graphene oxide was modified with ethylenediamine (EDA) by dissolving the EDA in ethanol and then adding equal parts of the resultant solution to a suspension of graphene oxide in DI water, resulting in a 1:1 ethanol–water mixture and a concentration of 1 mg/mL of both graphene oxide and EDA.^36^ The mixture was washed as above via centrifugation three times and redispersed in DI water.

### Monolayer Film Formation

Monolayer films were prepared on a silicon wafer using LB deposition with a KSV 2000 mini-trough with a temperature-controlled subphase kept at room temperature according to the usual procedure.^37^ HCl was added to the subphase bath until a pH of 2 for MXene and a pH of 4 for graphene oxide were achieved. All samples were bath sonicated for 3 min immediately before use. A suspension of materials (either graphene oxide or MXene) in 1:5 water and methanol at a concentration of 0.05 mg/mL was spread dropwise on the water’s surface and left to allow the organic solvent to evaporate for 30 min.

Compression isotherms were obtained at a rate of 5 mm/min to monitor the necessary surface pressure for monolayer formation without stacking and aggregation. Surface pressures for monolayer deposition were selected in disperse and condensed states based on isotherm data as indicated below. Langmuir monolayers were deposited onto silicon wafers at a rate of 1 mm/min at selected surface pressures. The silicon oxide surface of silicon wafers with local roughness of 0.1 nm within 1 μm × 1 μm surface areas is an atomically flat substrate, critically important for investigation of nanoscale interfacial properties. Samples were dried while remaining upright under ambient conditions.

## Characterization

### X-ray Photoelectron Spectroscopy

X-ray photoelectron spectroscopy (XPS) measurements were conducted using a Thermo K-α XPS instrument with Al Kα radiation (*h*ν = 1486 eV). Samples were prepared using drop casting onto silicon wafers to ensure appropriate thickness and coverage of the substrate. Survey scan spectra were taken three times with binding energies ranging from 0 to 1350 eV in 1 eV increments, and the high-resolution scans were collected ten times for each element in 0.1 eV steps. The obtained spectra were analyzed with Thermo Scientific Avantage Software.

### Optical Microscopy

Optical microscope images were collected with an Olympus BX51 microscope using the bright field reflection mode. Images were taken with a 10× objective lens.

### Attenuated Total Reflectance Fourier Transform Infrared Spectroscopy

Attenuated Total Reflectance Fourier Transform Infrared Spectroscopy (ATR-FTIR) was done with a Bruker Vertex 70 system by drop casting samples onto a silicon crystal substrate for ATR with a range of 4000–400 cm^–1^ and resolution of 2 cm^–1^. The resulting spectra were smoothed, background subtracted, and fitted in Origin software using known peak assignments.

### Atomic Force Microscopy

AFM topographical imaging was conducted using a Bruker Dimension Icon AFM in conventional light-tapping mode.^38^ Typically, scans were conducted using a probe with a nominal tip radius of 8 nm, a scan rate of 0.5 Hz, and a resolution of 512 × 512 pixels. Flake thickness was derived using the NanoScope software from height histograms and the average value of each peak.

Surface mechanical mapping was collected using a QNM in peak force tapping mode. The probe-sample interactions are translated into mechanical information about the sample. QNM was typically conducted with an RTESPA probe with a 40 N/m spring constant, a scan rate of 0.5 Hz, and a resolution of 512 pixels × 512 pixels. All AFM tips were similarly calibrated on a known material, sapphire, chosen for its hardness and lack of deformation during the tip–sample interaction with a sharp tip before measuring each sample. Ultimately, the indentation depth and load force were used for mechanical response evaluation. All measurements were kept consistent and calibrated with a hard sample (sapphire). Ultrasharp tips with a smaller radius of curvature were chosen for improved lateral resolution for surface mapping. Measurements are considered only with force applied appropriately in the linear regime, and the probe and cantilever spring constant were chosen with respect to the hardness of the samples at 40 N/m. Quantitative values were analyzed and calculated in NanoScope software by comparing substrate and single flake regime absolute values to display changes in properties in comparison with the known substrate. Values were taken from three different locations on each of the five different image scans.

The KPFM technique was used to map the surface potential distribution in noncontact, lift mode with frequency modulation.^38,39^ KPFM was typically conducted with a PFQNE-AL probe, which is conductive and has a spring constant of 0.8 N/m. Probes were tuned and calibrated with the same procedure as described in QNM. Scans were conducted with a rate of 0.5 Hz and a resolution of 256 × 256 pixels. Scans were kept consistent with a lift height of 70 nm above the sample surface for all samples. Images were collected for five different areas on each sample, and histograms of surface potential distribution were created.

Nano-IR imaging was performed using Bruker Anasys NanoIR3 AFM, which combines nanoscale infrared (Nano-IR) spectroscopy with AFM.^40^ The Nano-IR 3 employs a photothermal response to accurately map the chemical composition and material properties with exceptional spatial resolution. When infrared light is absorbed at a particular wavelength, it induces local microscopic thermal expansion, which, along with changes in surface stiffness, is detected with tip deflection. Nano-IR mapping provides localized chemical information that can be derived from thermal expansion after being irradiated by a laser beam.^18^ Spectra were taken during illumination from 800 to 1800 cm^–1^ at a resolution of 4 cm^–1^. Chemical mapping images can be obtained at a selected characteristic single wavenumber chosen from IR signature with a resolution of 512 × 512 pixels and a scan rate of 0.5 Hz.

Ten spectra were taken per sample on locations indicated to be the monolayer thickness of the samples. Additional samples were fabricated via drop casting to eliminate substrate interference, and ten spectra per bulk area were taken for each for comparative purposes. For all samples, five images were taken at different locations for each specific wavenumber of interest. All spectra were analyzed in Origin with smoothing and peak assignment. Height and IR profiles were analyzed using Gwyddion software to acquire profiles horizontally on the image plane at the exact same line value in comparative images.

## Results and Discussion

### LB Monolayers

As is known, neither graphene oxide nor MXene is sufficiently amphiphilic to ensure the stability of the film on the water surface.^41^ Attempts at fabricating reproducible Langmuir monolayers with only graphene oxide or MXene were not successful.^42^ Therefore, the experimental procedure has been altered with either the addition of an amphiphilic molecule as a surfactant or by modification of the subphase to alter the charge distribution on the 2D flakes to allow them to form a stable monolayer at the air–water interface.^43^

Lowering the pH of the bath resulted in a decrease in the surface charge of the flakes, which improved their stability at the air–water interface. This occurs both with the MXene and the graphene oxide flakes due to the presence of surface–OH groups.^44^ We found that the addition of hydrochloric acid (HCl), resulting in a pH of 2–4, drastically improved the film uniformity and stability for multiple hours. Overall, stable and reproducible pressure isotherms were collected under lowered pH subphase conditions suggested above.

Compression isotherms showed a gradual increase in surface pressure, followed by a more rapid slope change as the available surface area decreased (Figure 2). Large hysteresis was observed during the first compression, and the second cycle showed a reproducible shape with minimum hysteresis, a usual behavior for stiff 2D monolayers.^45^ AFM images were collected for monolayers deposited at 9, 13, and 19 mN/m for graphene oxide samples and at 30, 20, and 10 mN/m for MXene samples, representing different monolayer states from dispersed to densely packed (Figures 2 and S1).

**Figure 2:**
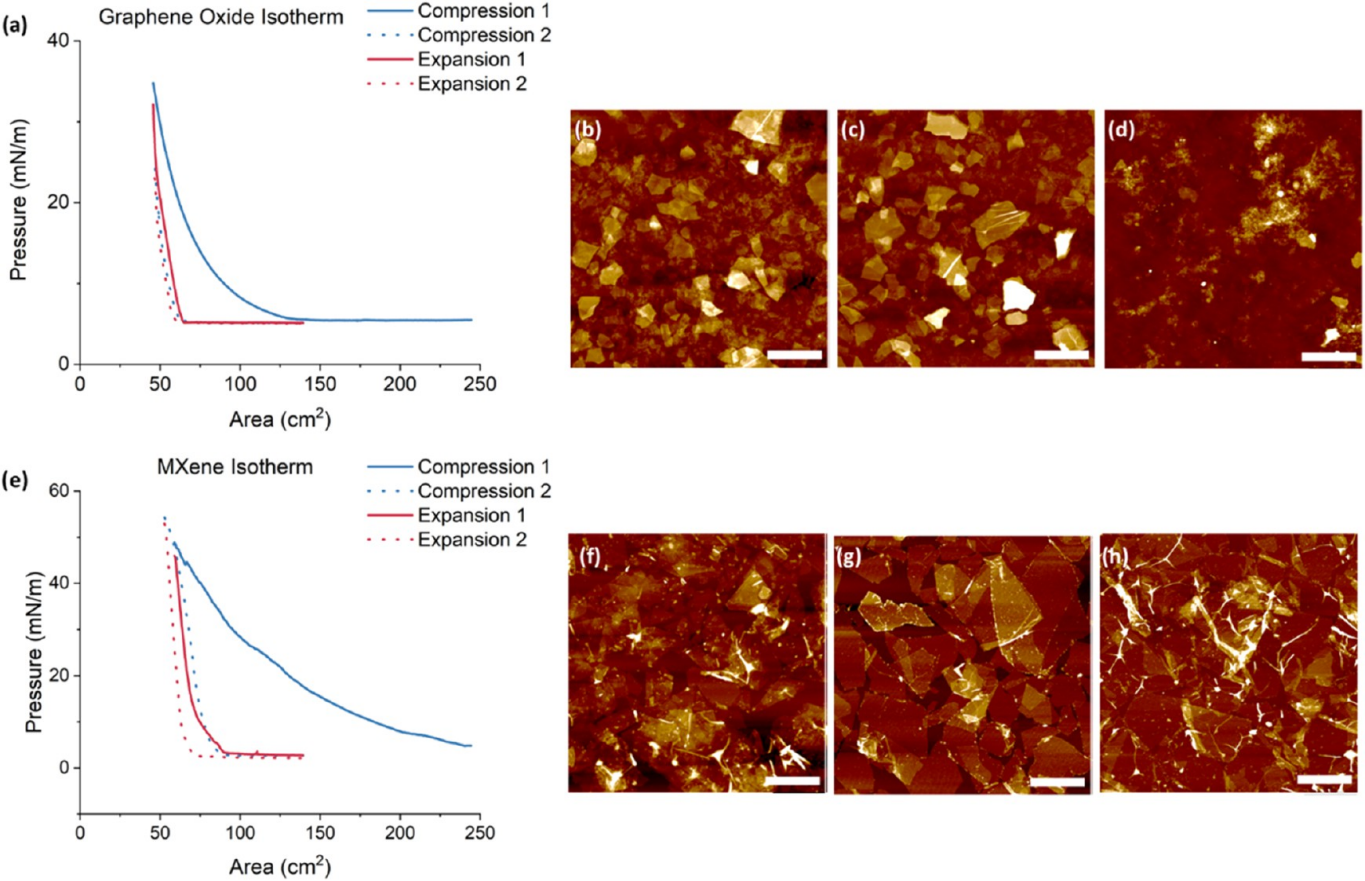
Formation of Langmuir monolayers at the air–water interface and surface morphologies of the deposited monolayers at different surface pressures. The graphene oxide pressure–area isotherm at a pH = 4 and corresponding AFM images (a–d); MXene pressure–area isotherm at a pH = 2 and corresponding AFM images (e–h). LB monolayers deposited at a pressure of (b) 19 mN/m, (c) 13 mN/m, (d) 9 mN/m, (f) 30 mN/m, (g) 20 mN/m, and (h) 10 mN/m, respectively. The lateral scale of all AFM images is 2.0 μm. The z-scale for AFM images is 70 nm (a, b), 30 nm (c), 50 nm (f), and 25 nm (g, h).

AFM images of graphene oxide flakes at different surface pressures show differences in flake morphology and dispersity. At the lowest surface pressure, large areas of the silicon substrate are visible among dispersed flakes (Figure 2d,h). The surface density of packing of the graphene oxide flakes increases until they are no longer distinguishable and fully overlapping at high surface pressure (Figure 2c–e).^37^ At the highest pressure, significant agglomeration is evident, and characteristic flake defects show up due to crumpling and folding (Figure 2b,f). The intermediary pressure for both samples results in high packing density without excessive overlapping and distortion of the flakes and thus can be used for further mapping with high resolution (Figure 2c,g).

Optical microscopy confirms the appearance of surface coverage at the optical scale as observed at a submicron scale with AFM (Figure 3). We observed that graphene oxide monolayers show diverse surface coverage with varying uniformity and larger aggregation after modification with EDA. Monolayers are preferential in areas with larger flakes, and agglomerates tend to form with increasing flake defects and decreasing sizes. On the other hand, MXene monolayers show a highly dispersed optical appearance (Figure 3). Finally, modified MXene monolayers exhibit lower surface coverage on a large scale but with occasional larger local agglomerations.

**Figure 3:**
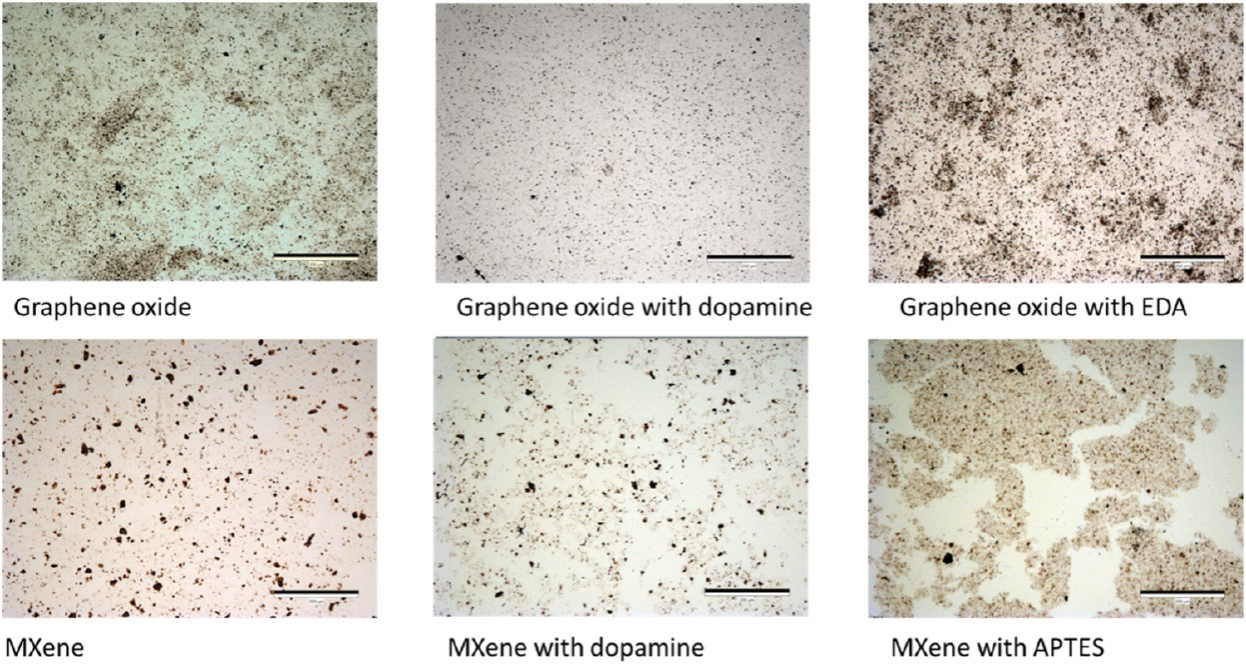
Optical images of LB films, graphene oxide without and with surface modification with corresponding surface pressures of 13, 15, and 15 mN/m, respectively (top), and MXene without and with surface modification with corresponding surface pressures of 20, 12, and 10 mN/m, respectively (bottom). The lateral scale bar for all images is 200 μm.

### Flake Modification and Effect on Monolayer Formation

XPS survey spectra of modified 2D materials confirm an expected change in their surface chemical composition (Figure 4). For the modified graphene oxide, one can see that the C–O bonds decrease in comparison to the C–C bonds, indicating binding with the organic molecules and a loss of available initial hydroxyl surface groups. Similarly, for the modified MXene sample, one can see a gain of C–C bonding that confirms the covalent binding of the organic molecules.

**Figure 4:**
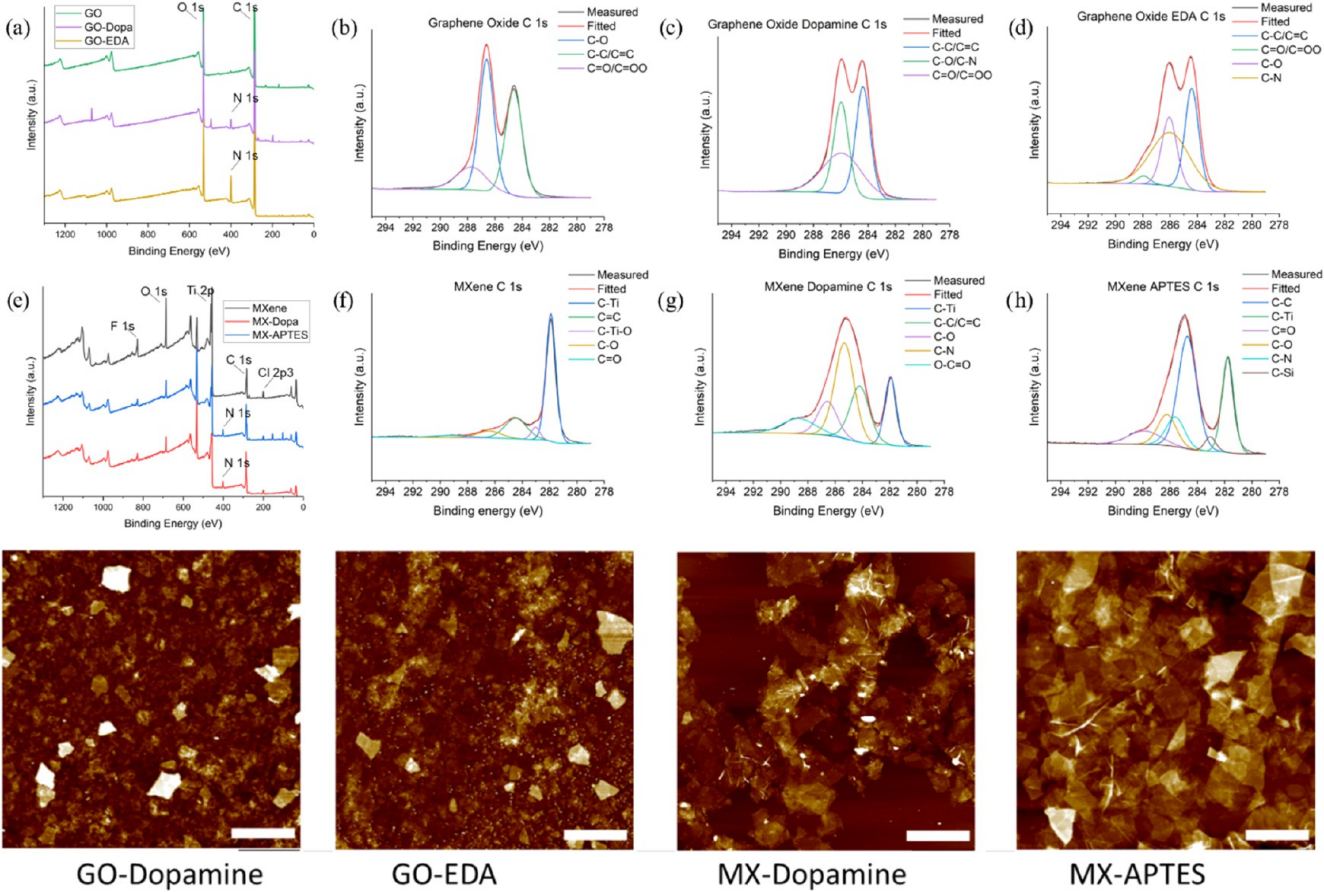
XPS of functionalized graphene oxide samples (a–d) and MXene samples (d–h). Corresponding topographical AFM scans for different monolayers (h–k). Z-scale bar for AFM images is 50 nm (i–k) and 100 nm (l). Lateral scale bars for all are 3.0 μm. Corresponding surface pressures are indicated in Figure 3.

There is also an indication of gained C–N bonds due to the presence of dopamine and APTES. Overall, smaller flakes were observed after modification due to the additional centrifugation and sonication steps. Moreover, AFM images show an increase in the average flake thickness after chemical modification (Figures 4 and S1).

Flake thickness can be seen to double in the case of modified MXene and quadruple in that of graphene oxide, both of which gain over 4 nm of thickness (Table S2). In one specific case, MXene flakes were functionalized with dopamine at 1:2, 1:1, and 2:1 mg/mL ratios to monitor flake thickness variation (Figure S2). When individual portions of modified flakes are examined, dopamine concentration appears to be full coverage throughout, independent of concentration as indicated by the definitive height difference on the entire surface of the flake compared to an unmodified flake. The flake height becomes more homogeneous with increasing the ratio of dopamine hence why the sample at a 1:1 ratio was chosen to provide full coverage (Figure S2). Additionally, the presence of surface modification can be seen with the change in surface texturing of the modified flake (Figure S2).

Variable thicknesses at different concentrations are evident for dopamine functionalization with flakes modified at various pH conditions. At higher dopamine concentrations, the flake thickness increased from an average height of 6.3 ± 2.5 nm to 10.5 ± 1.5 nm and 30.2 ± 2.1 nm, respectively (Figure S2). Surface morphology changes are also apparent, with higher concentrations inducing a larger agglomeration of dopamine-functionalized flakes. The intermediate concentration provides complete coverage of each flake as can be seen in the height profile analysis of the modified flakes (Figure S2).

### Nanoscale Chemical Composition Mapping with Nano-IR Mode

As we know, the Nano-IR mode has a spatial resolution of 10 nm over the fingerprint region of the IR spectrum and has proven to be instrumental for mapping the chemical composition of thin polymer films and monolayers.^18,46^ It is important to compare Nano-IR with bulk FTIR measurements to confirm Nano-IR signature and selection of proper bands (see example for dopamine (Figures S3 and S4)).^47^ Next, to distinguish IR peaks of interest in Nano-IR spectra, substrate contributions were first considered. Drop-cast samples are compared to LB monolayers to ensure the minimization of substrate influence and indicate the reliable detection of characteristic IR peaks independent of the substrate (Figure S5).

For instance, graphene oxide modified with EDA shows a sharp IR peak at 1014 cm^–1^, due to the C–N stretching (Figure S5b). This peak is also present in the silicon wafer corresponding to the Si–O bonds. The peaks at 1066 and 1114 cm^–1^ in the FTIR data are seen in all three samples, with the EDA modification causing a peak shift to a higher wavenumber (Figure S5a–c). In the Nano-IR data, these peaks are no longer present. Instead, a minor peak shift in the EDA-modified sample to 1120 cm^–1^ is visible. At 1400 cm^–1^, the unmodified graphene oxide peak in the FTIR measurement is present, which corresponds again to a peak shifting with Nano-IR.

The functionalization of graphene oxide with ethylenediamine includes covalent bonding between the nitrogen atoms and the carbon of graphene oxide (Figure 1).^12^ This includes a gain of C–N bonds and a loss of N–H bonds. Both the peak at 1014 cm^–1^ that appears after modification and that at 1114 cm^–1^ are attributed to the additional C–N stretching (Figure S5). The peak at 1096 cm^–1^ is due to C–O stretching of the graphene oxide lattice. The loss of the peak at 1580 cm^–1^ can be attributed to the loss of N–H groups. The peak at 1634 cm^–1^ on the conventional FTIR spectrum is due to the C=C stretching in the graphene oxide in both unmodified and modified samples and slightly shifted to 1600 cm^–1^ on the Nano-IR spectrum.^48^

After the analysis of Nano-IR spectra collected and identification of characteristic IR bands in comparison with common FTIR signature, mapped images at characteristic C=C stretching at 1600 cm^–1^ were taken of modified graphene oxide (Figure 5). From the mapping of EDA-modified flakes, we can conclude the presence of amine bonding is apparent throughout the surface of the modified flakes, confirming full surface modification. Areas of higher intensity congregate on the edges of the flakes, demonstrating higher concentrations of EDA and preferred bonding at the edges of the flakes.

**Figure 5:**
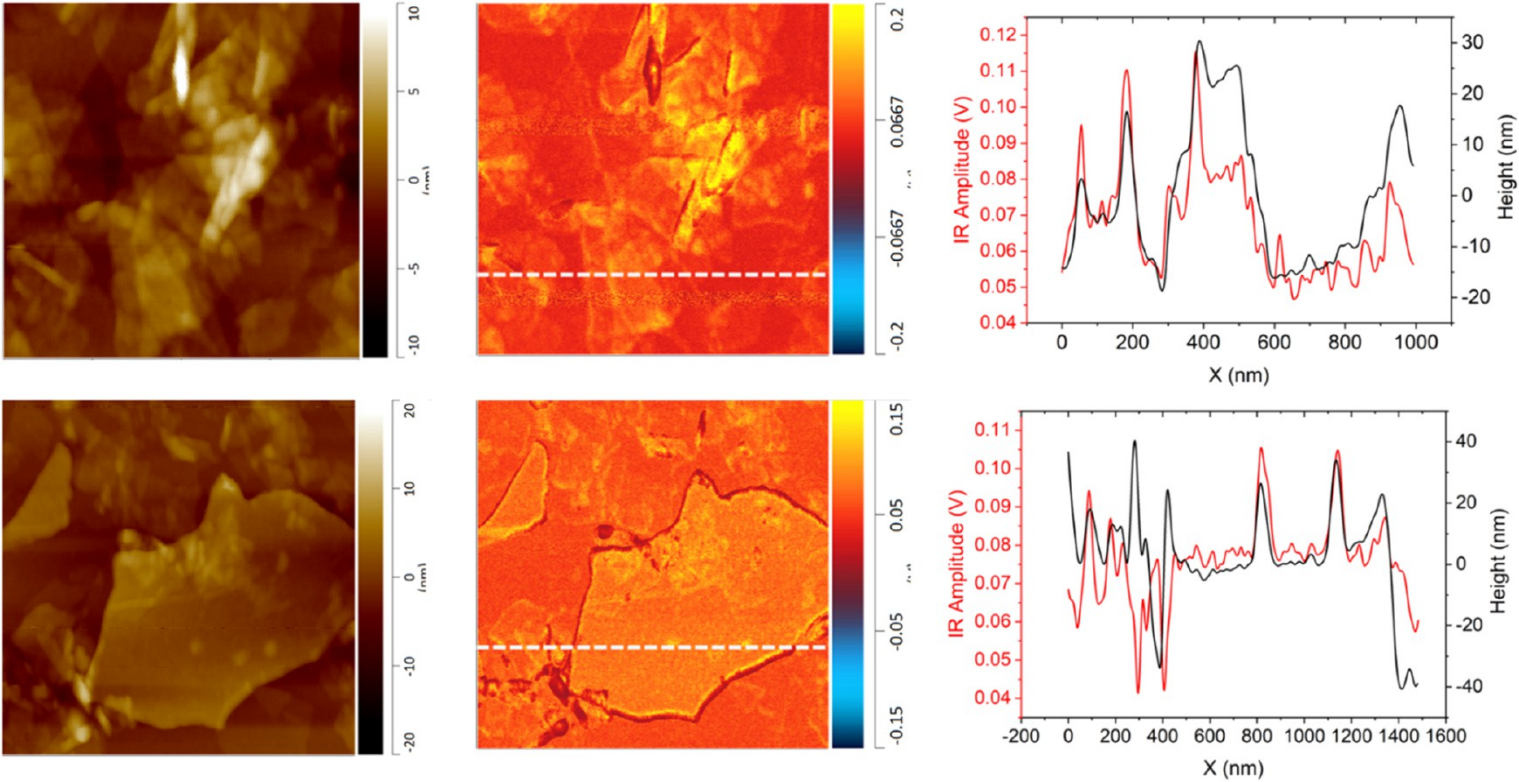
Topography (left) and nano-IR mapping (right) of modified graphene oxide at 1600 cm^–1^ with corresponding profiles of modified flakes. Graphene oxide was modified with EDA (top) and dopamine (bottom). Lateral image sizes are 1 μm × 1 μm.

The functionalization of graphene oxide with dopamine includes covalent bonding as well as interactions between both molecules’ −OH groups, creating a loss of O–H bonds and a gain of C–O bonds as indicated by the appearance of characteristic peaks.^17^ Indeed, similarly to EDA-modified flakes, peaks at 1096 and 1634 cm^–1^ are present for dopamine-modified graphene oxide flakes. In addition, peaks at 1500–1522 cm^–1^ are due to the C=C stretching from the benzene ring of the dopamine. The peak at 1096 cm^–1^ from the graphene oxide is shifted lower with the addition of dopamine. The peak at 1634 cm^–1^ from the graphene oxide flakes is now at 1600 cm^–1^, which can be seen in the bulk dopamine and is another contribution from the C=C stretching of the amine ring bound to the surface.^14^

Overall, chemical surface mapping of a modified flake shows the presence of dopamine coverage across the whole flake surface. Moreover, mapping reveals the preferential bonding to the flake edges, likely due to a larger increase of defects on the edge of the structure, similar to those observed for EDA-modified flakes (Figure 5). There are a few local areas of increased presence of the EDA throughout the flake’s surface as well, most often forming in the vicinity of surface corrugations and overlapping regions of neighboring flakes.

In contrast to graphene oxide materials, pristine MXene flakes do not show a strong IR response. As is known, the functionalization method includes covalent bonds between both molecules’ −OH groups, creating a loss of O–H bonds and a gain of Ti–O bonds.^49,50^ And this additional bonding does not show IR activities within the fingerprint range of Nano-IR measurement. The peak at 1014 cm^–1^ is only present on the dopamine-modified thin sample, which could correspond to the substrate contribution (Figure S8a,c). However, since it is not present on the thin unmodified MXene, it can be attributed to C–N stretching.

A minor peak at 1507 cm^–1^ only prominently appears in the dopamine-modified MXene, can be attributed to C–N stretching, and corresponds to a strong peak on the bulk dopamine IR spectrum. A peak at 1600 cm^–1^ can be related to C=C stretching in the peak due to the amine contribution.^49^ In the FTIR spectrum, this peak at 1634 cm^–1^ is not present in the unmodified sample (Figure S8c). The unmodified MXene shows a peak at 1733 cm^–1^ corresponding to the C=O stretching. These two peaks in the Nano-IR spectra are easier to discriminate between in the thin samples. A summary of all peak assignments is presented in Table 1.

**Table 1:** Peak Assignments for Different Peaks in FTIR and Nano-IR Spectra.^14,47,48,50^

peak (cm^–1^)	assignment
1014	C–N
1040	Ti–O–Si, Si–O
1096	C–O
1114	C–N
1410	Si–CH_2_
1500–1522	C=C
1580	N–H
1600	C=C
1634	C=N
1733	C=O

The peak at 1600 cm^–1^ can be attributed to the dopamine bonding to the MXene surfaces (Figure 6, compare to pristine MXene in Figure S9). The IR mapping at this wavenumber shows that there is a higher density of dopamine at the center of the flake, in contrast to modified graphene oxide flakes with preferential edge bonding. In addition, the IR mapping at 1422 cm^–1^ shows the same surface patterning in the same locations on the flake’s surface. These peaks indicate dopamine bonding and verify that the chemical composition indicates the covalent bonding of dopamine. It is worth noting that for dopamine modification at a pH of 10.5, preferential binding to the microscopic defects and near the flake edges is observed, which partially compromises full flake surface coverage (Figure S9). Unlike basic conditions, binding at a neutral pH of 6.5, dopamine does not preferentially concentrate near edges (Figure 6).

**Figure 6:**
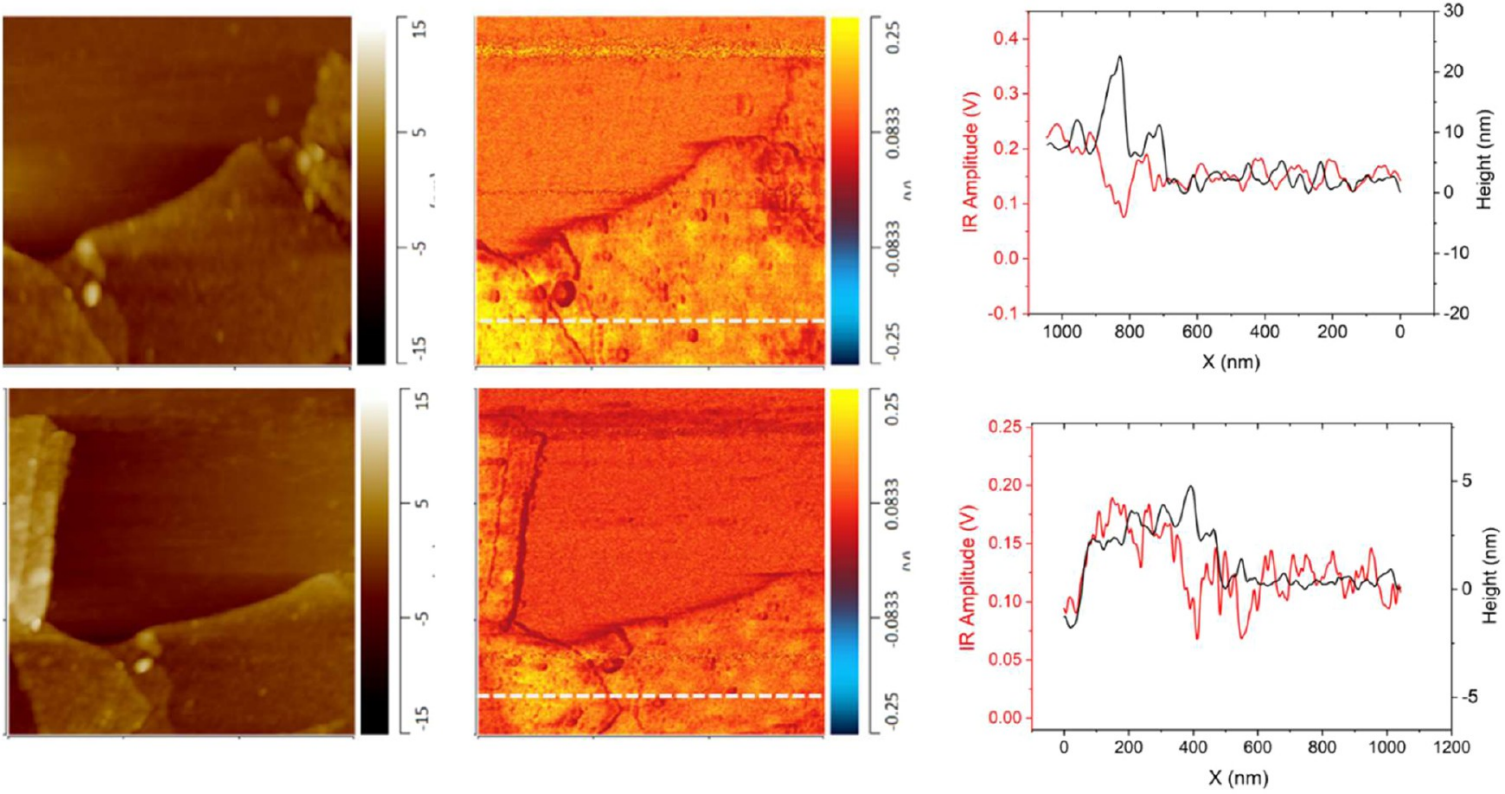
Nano-IR mapping of MXene modified with dopamine at wavenumbers 1600 cm^–1^ (top) and 1422 cm^–1^ (bottom). Height and IR amplitude profile along the flake surface (right). Lateral scale bar is 1 μm for all, height scale bar is 30 nm, and IR intensity scale bar is 0.5 V.

The overlay of IR amplitude and height profiles shows decoupling of the height and IR signal over the surface of the flake (Figure 6). There are distinguishable differences between the height and IR signal, as can be seen in their profile, where surface topography is relatively smooth as compared to IR amplitude, indicating dominating changes in variation in concentration of dopamine molecules and not surface features (Figure 6). On the other hand, some edge effects can be seen around the flake edge elevations. Indeed, it is evident at the 800 nm location of the 1600 cm^–1^ scan, whereby the IR signal decreases dramatically due to a lower contact area between the probe and sample as the edge of the second layer of a flake (Figure 6).

Next, MXene flakes modified with APTES show a peak of prominence at 1634 cm^–1^ (Figure 7). Appearance of 1634 cm^–1^ indicates the C=N bonding because of the addition of the chemically bonded organic layer. Finally, the presence of Si–CH_2_ deformation is indicated by the appearance of 1410 cm^–1^ (Figure S8b). For this functionalization scheme, the related bonding mechanism includes an increase in the formation of Ti–O–Si bonds and a loss of O–H bonds.^50^

**Figure 7:**
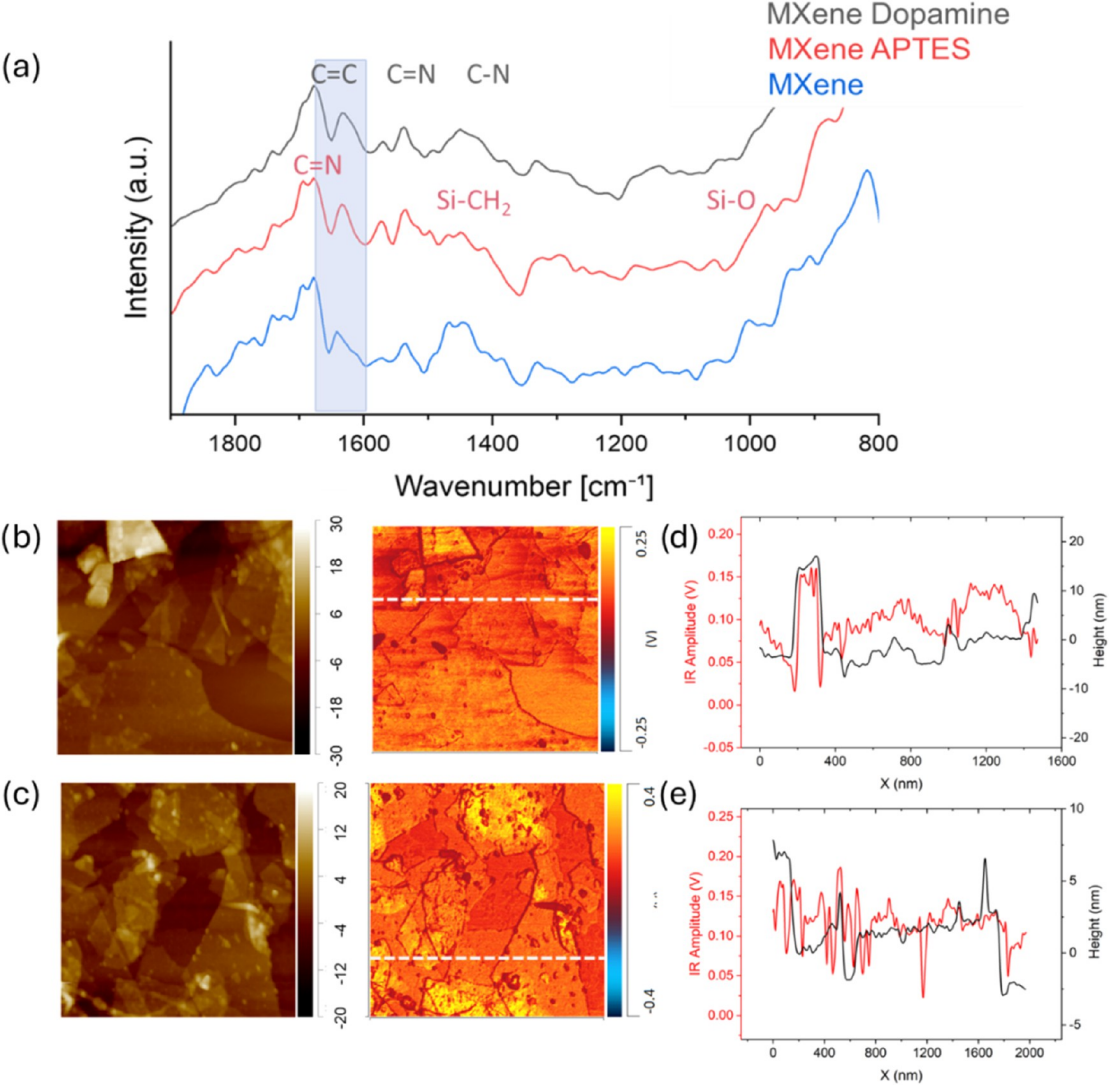
Comparison of MXene nano-IR spectra with the key area of interest labeled. All spectra are collected from flake monolayer films (a). Nano-IR mapping for MXene modified with APTES at wavenumbers 1014 cm^–1^ (b) and 1600 cm^–1^ (c). Lateral scale bars are 1.5 and 2 μm, respectively. Corresponding comparative height and IR intensity profiles of each image were collected at 1014 cm^–1^ (d) and 1600 cm^–1^ (e).

The nano-IR mapping at 1634 cm^–1^ (C=N bonding) shows IR intensity variation across the surface of the modified flake that is different from the uniform distribution on pristine MXene flakes (Figures 7 and S7d). The Nano-IR mapping shows comprehensive flake surface coverage of the APTES across the whole surface, with less presence of APTES at the edges of the flakes. Additionally, the peak at 1040 cm^–1^ can be attributed to a contribution from the silicon substrate itself.

It is worth noting that due to the nature of the functionalization routine, all flakes are modified in mixed suspensions before film formation at the air–water interface. Therefore, it is expected that multilayers of flakes will show a significant increase in the IR response due to increased concentrations of bonded molecules, in contrast to the individual modified monolayers because of penetration of IR radiation and loading depth. Comparative height and IR profiles show a proportional increase of IR response with total thickness increase, confirming functionalization of individual flakes within stacked aggregates (Figure 7d).

Overall, in comparison to dopamine-modified MXene, the APTES-modified MXene flakes show more uniform surface coverage after functionalization. There is a lower APTES concentration near the edge of the flake on both modified MXene flakes compared to the modified graphene oxide, with relatively uniform coverage. Varied signal intensity with changes in surface concentration is evident from intense 1014 cm^–1^ peaks, confirming the presence of APTES aggregates. However, overall flake surface coverage is uniform and complete for all surface areas of individual flakes, with higher APTES coverage observed at overlapped flake areas (Figure 6).

### Nanomechanical Surface Mapping

Next, after nano-IR chemical mapping, combined topographical and nanomechanical properties, such as concurrent topography, elastic modulus, and adhesion, were collected for individual flakes with different modifications. Adhesion from QNM measurements reflects the pull-off forces for silicon tips, and modulus reflects the loading force resistance measured during the elastic regime of compressive deformation. Pull-off forces are controlled by a combination of physical, mechanical, and chemical forces. Therefore, changes measured are expected due to flake chemical modification as well as the structural realities of individual flakes. Since a single layer of flakes is between 2 and 5 nm, the silicon substrate’s effect on apparent elastic modulus can be significant and cannot be easily deconvoluted.^51^ Indeed, from measurements of pristine MXene flakes, the apparent elastic modulus was estimated to be around 330 GPa, whereas graphene oxide flakes show an apparent elastic modulus reported at 200 GPa that far exceeds the limits of measurement with silicon tips and thus will not be discussed further.^2,52^

A shadow effect is present in the modulus of thicker samples, such as graphene oxide modified with dopamine (Figure 8c). Edge effects are negligible in the MXene images due to the thinness of the flakes, and no sharp change in modulus is evident (Figure 9). However, in contrast to the substrate-affected artificial elastic modulus, the surface adhesion can be estimated and compared to the surrounding silicon surface areas (Table 2). Therefore, we are able to form quantitative comparative measurements of varied flakes under similar probing conditions. In order to avoid the edge contributions, all adhesive data were acquired at central areas on the flakes. Edge effects are inevitable due to the tip dilation as visible on areas with significant height variation.^53^

**Figure 8:**
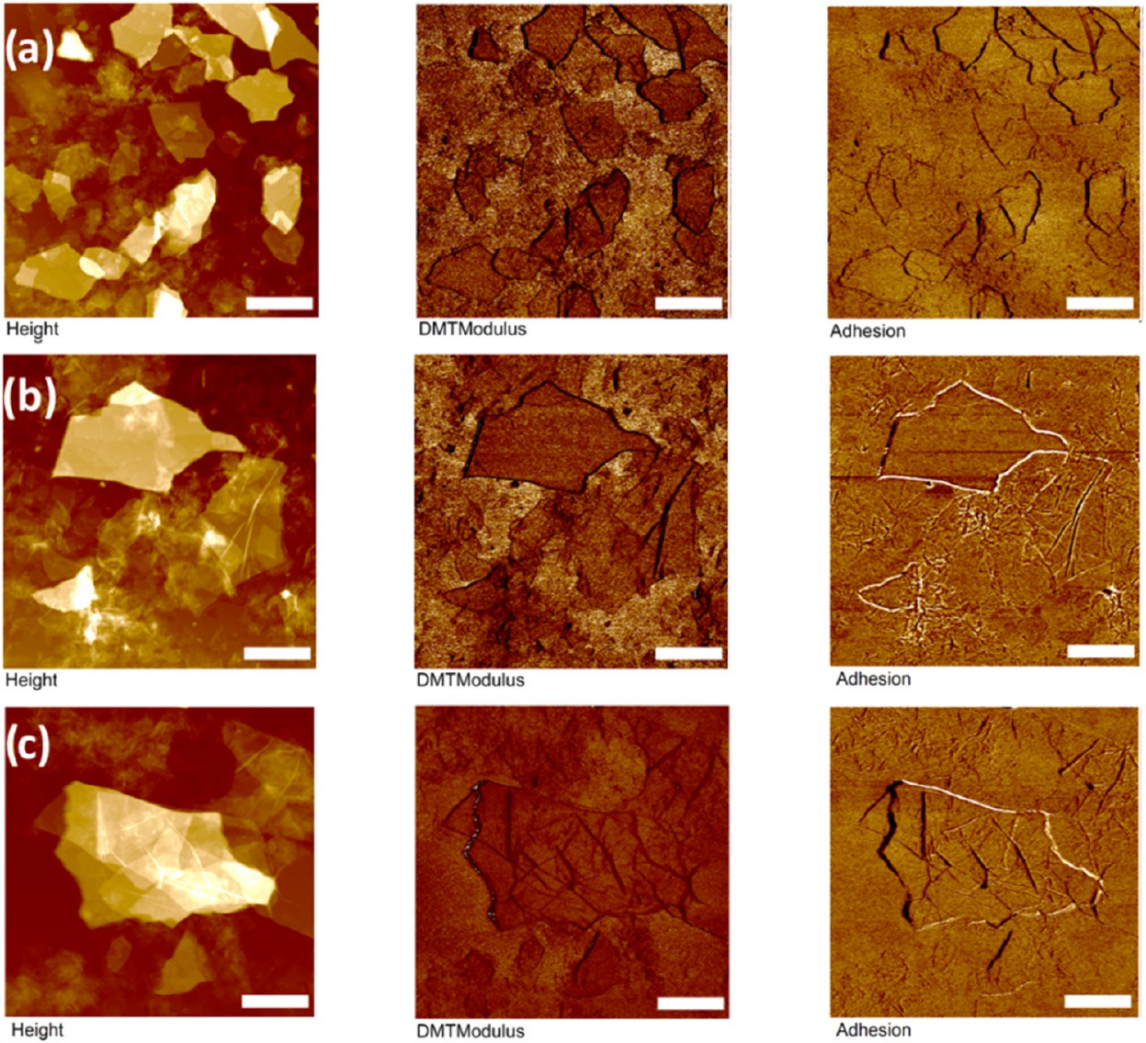
QNM images of graphene oxide flakes. The columns are the height (left), modulus (center), and adhesion (right). The samples are graphene oxide with (a) no modification, (b) modified with EDA, and (c) modified with dopamine. The z-scale bar for height is 50, 70, and 160 nm, respectively, for all moduli is 50 GPa, and for all adhesion is 12 nN. Lateral scale bar is 1.0 μm (a, c) and 700.0 nm (b).

**Figure 9:**
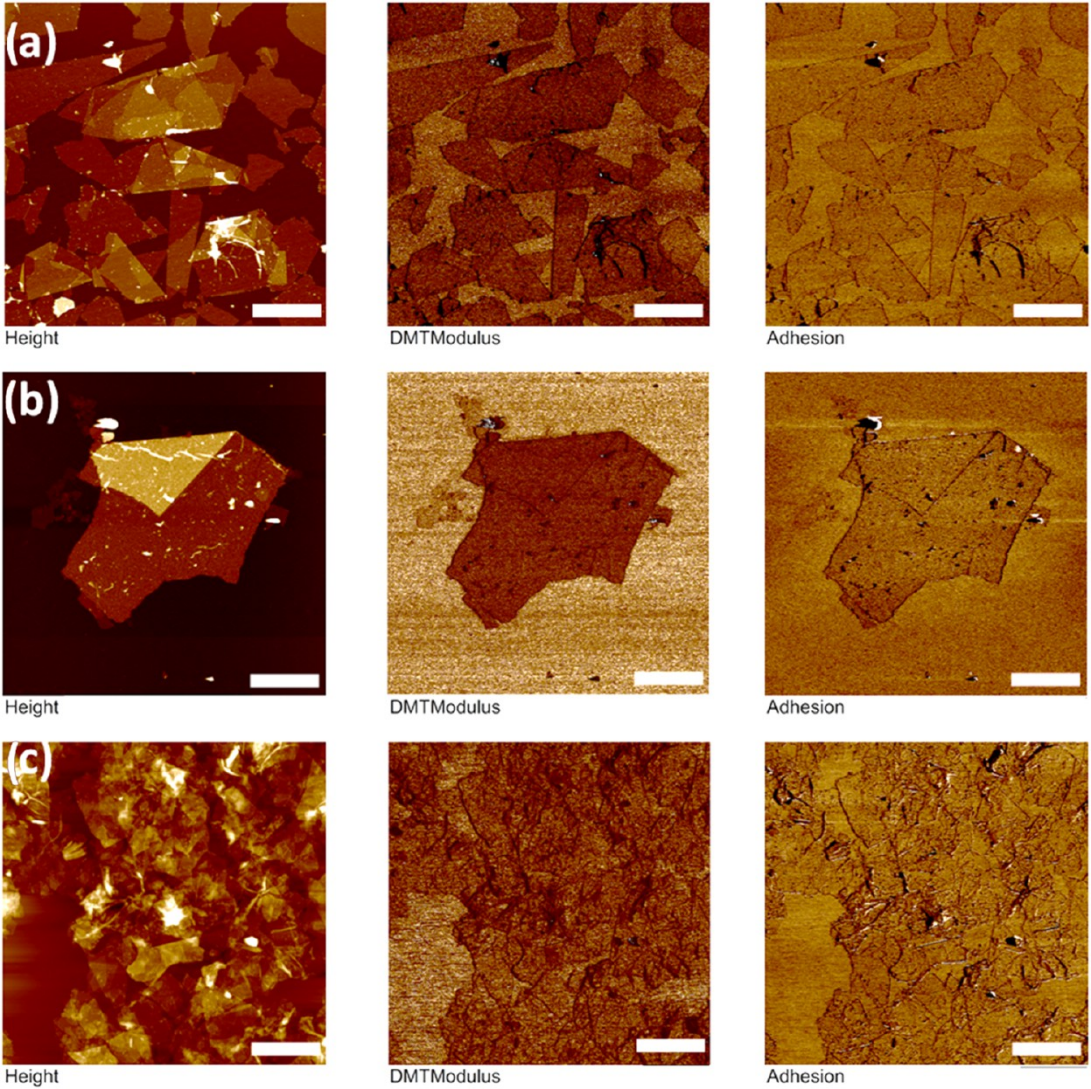
QNM images of MXene. The columns are height (left), modulus (center), and adhesion (right). The samples are MXene with (a) no modification, (b) modified with dopamine, and (c) modified with APTES. The z-scale bar for height is 25, 40, and 90 nm, respectively, and the scale bar for modulus is 40 GPa (a) and 80 GPa (b, c) and for adhesion is 12 nN for all panels. Lateral scale bar is 2.0 μm for all.

**Table 2:** Difference in Adhesion for Flake Surface in Comparison to the Surrounding Substrate

sample	relative change in adhesion (nN)
GO	+1.1 ± 1.1
GO dopamine	+0.9 ± 0.6
GO-EDA	+0.9 ± 0.7
MX	+0.4 ± 0.3
MX dopamine	+1.8 ± 0.9
MX-APTES	+1.2 ± 0.7

As was observed, the adhesion does not change (within statistical deviations) after modification of graphene oxide flakes with pre-existing rich surface chemistry with a variety of polar and hydrogen binding surface groups (Table 2). In contrast, the adhesion increases significantly after modification of MXene flakes, conforming to a dramatic alternation of surface composition from relatively surface composition in pristine flakes (Table 2). Though both types of flakes are amine-modified, Nano-IR shows a 2-fold increase in the relative concentration of amine groups due to higher grafting density on reactive sites (Figure 5). This increase in amine surface concentration causes dramatic increase in adhesive forces to Si–OH surface groups of the silicon tip. Via Nano-IR measurements, we have shown that there is high surface coverage of organic molecules on the surface of these flakes with varying degrees of uniformity across the flakes. The increasing surface coverage results in increased effective surface roughness (Figure S2). This topographic change also can indicate stronger adhesion due to the heterogeneous surface, leading to increased contact area with the AFM probe. It is worth noting that monolayer films maintain stability over the course of a few months of storage and the change in surface mechanical properties was not noticeable in our studies.^7^

Next, QNM images show “apparent” modulus and adhesion distribution, which are tightly correlated over the flake surfaces and changes due to defects, wrinkles, edges, and aggregated modifications (Figure 8).

Graphene oxide flakes show uniform surface distribution for single- and double-stacked flakes with some difference observed for larger stacks. Graphene oxide is relatively stable under ambient environmental conditions, and there is no evidence of surface stiffness degradation after chemical modification other than some variability in appearance due to sonication and centrifugation. Overall, the apparent modulus is reduced on staggered flakes, and a higher adhesion is observed along flake edges with an excessive amount of modifying agents.

On the other hand, MXene shows higher surface heterogeneity due to the reduced stability during the chemical modification process (Figure 9). It is worth noting that minor MXene oxidation is present in the pristine sample due to titanium oxide nanoparticle growth across the flake surface with apparent reduced elastic moduli (Figure 9a).^54^

MXene modified by APTES shows that the surface texture has changed with the lower modulus and adhesion at molecular aggregates with significantly lower modulus and adhesion (Figure 9c). The adhesion also significantly decreases across the surface areas in close proximity to the edges of the flakes, indicating some depletion of surface functionalities in these areas. Finally, we observed a subsequent sharp increase in apparent adhesion across the edge of the flake, which is likely due to the tip-edge side interactions.^55,56^

### KPFM Surface Potential Mapping

Next, KPFM measurements have been conducted to determine the surface potential distribution across pristine flakes and modified flakes as contact potential difference under electrical field applied in a noncontact mode to identify the role of molecular dielectric layer (Figure 10).^57^ KPFM has been used to show that the reduction of graphene oxide due to high laser irradiation that the surface potential of graphene oxide but KPFM scanning are stable.^58^

**Figure 10:**
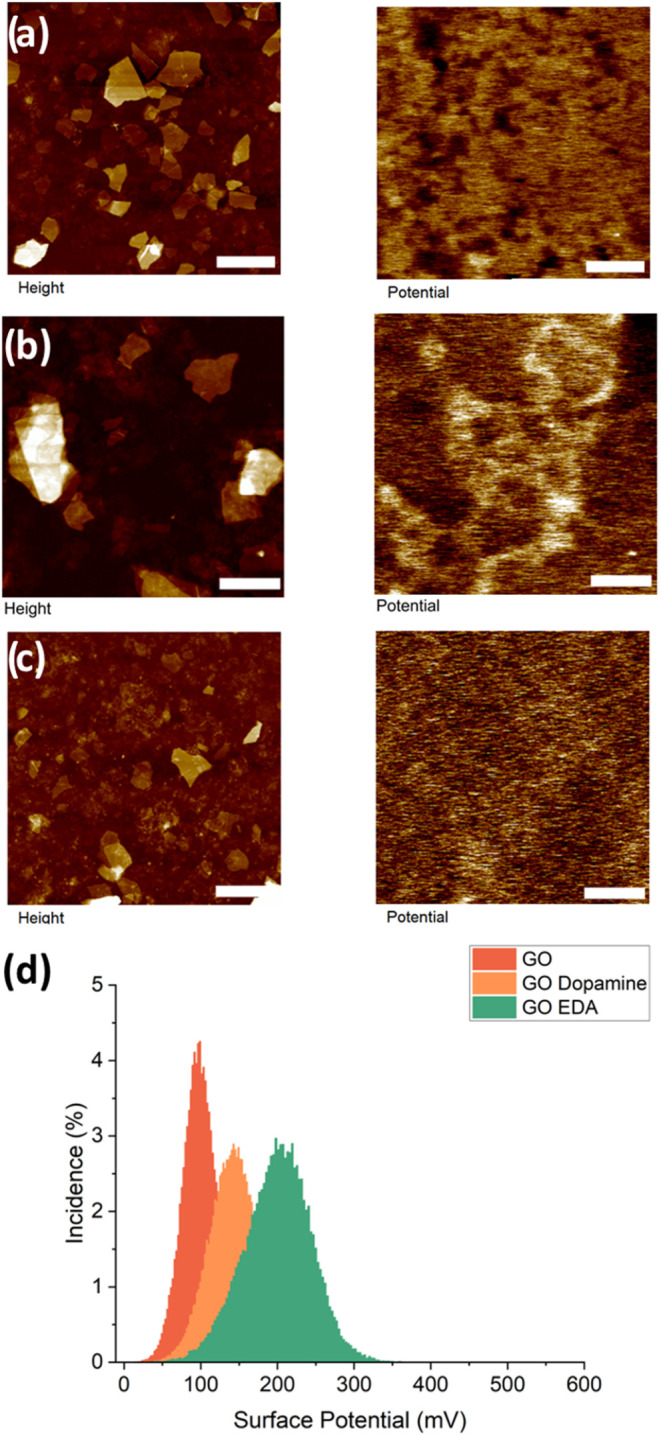
KPFM surface potential mapping of graphene oxide. The left column is topography, and the right column is surface potential with a scaled *z*-axis for comparison of the six samples. The samples are graphene oxide with (a) no modification, (b) EDA, and (c) dopamine. Representative histograms (d) of surface potential. The vertical z-scale is 50, 110, and 60 nm for topography (a–c), and the z-scale for surface potential is 200.0 mV (a–c) and 250.0 mV (b). Lateral scale bar is 2.0 μm for all.

Comparing surface properties over the extent of the scan using histogram data can show how the surface potential changes with flake modification. As is known, the electrostatic force acting on the tip–surface pair can be expressed as

$$F=\frac{1}{2}\frac{\delta C}{\delta z}V^{2}$$

where $\frac{\delta C}{\delta z}$ is the distance-dependent capacitance gradient between the sample and probe, *C* is the capacitance, *C*, is proportional to dielectric permittivity, ε: *C* ∼ ε, and *V* is the contact potential difference.^59^

Overall, one can see the rise of the surface potential average with the addition of amine functionalization on both graphene oxide and MXene flakes (Figures 10d and 11d). It is worth noting that the broad distribution of surface potential masks contributions from different surface areas (visible in some images in Figures 10 and 11) and shows only averaging across the whole image.

**Figure 11:**
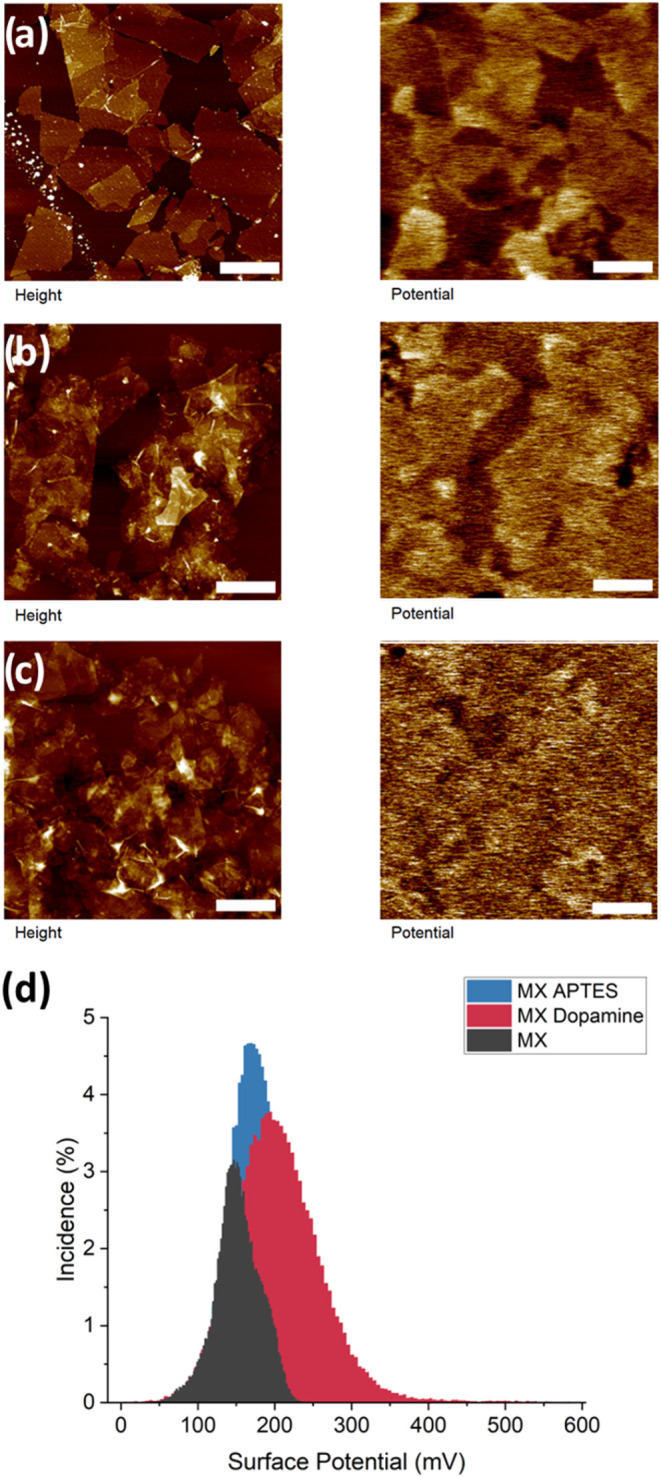
Surface potential mapping via KPFM of MXene. The left column is topography, and the right is surface potential with a scaled *z*-axis for comparison of the six samples. The samples are MXene with (a) no modification, (b) dopamine, and (c) APTES. Representative histograms (d) of surface potential. The vertical z-scale is 20, 45, and 70 nm for topography (a–c), and the z-scale for surface potential is 200.0, 400.0, and 200.0 mV. Lateral scale bar is 2.0 μm for all.

As we observed, dopamine coating results in an increase of the average surface potential by 44% for modified graphene oxide and 31% for MXene flakes (Table S3). This can be further ascribed to the amine surface layer presence due to the increased total capacitance caused by the increased effective dielectric constant in the tip-flake gap. with an added dielectric layer in between. Next, a further increase of the surface potential was observed for the graphene oxide modified with an EDA surface layer. Finally, APTES, which presents an alternate example of functionalization, consequently, increases the modified MXene surface potential as well, though not to the same extent as does the dopamine layer. As is known, surface potential is correlated to work function, thus assuring that the presence of electron-rich groups such as NH_2_ tend to raise work function and thus surface potential observed in KPFM mapping.^60^

The surface potential remains uniform over each flake and between each flake, independently of stacking and aggregation. Surface potential contrast of graphene oxide flakes can be compared to the silicon substrate (Figure 10a). Modified graphene oxide flakes show much lower contrast, and, however, they maintain uniformity independent of flake morphology (Figure 10b,c). Among all samples, the dopamine-modified graphene oxide flakes have the lowest surface potential contrast.

Comparing individual locations on the surface potential maps can show the ways in which surface potential changes for flake-to-flake disparities. It is known that increasing the layers of graphene does not necessarily improve their surface potential due to different dopant conditions.^61^ Therefore, it is not unexpected that there is lowered surface potential where there are areas of stacked flakes (Figure 10). This can also be extrapolated to similar phenomena occurring with MXene stacks. Comparatively, the MXene samples show more distinct contrast with the substrate in surface potential, in addition to higher values (Figure 11). For a comparison of all data for pristine and modified flakes, see Table S2.

The higher potential areas do not merely correspond to the thicker stacked flake areas. Indeed, places where the flakes are folded in on themselves show lower potential than flattened flakes. Scaled potential shows a significantly higher contrast of the MXene flakes overall. Dopamine-modified MXene flakes show 2-fold higher surface potential due to the presence of amine groups (Figure 11b). Additionally, MXene flakes have distinct potential maps that show decreases in potential at folds and uneven areas. APTES modification resulted in the uniform surface potential within MXene flakes (Figure 11c).

Overall, we suggest that the apparent increase in surface potential can be associated with the additional dielectric layer that increases effective capacitance. Dielectric constants for all organic compounds explored in this work are within a range from 9 to 15 (see Table S2).^62,63^ Overall, we observed that the presence of an organic layer with a higher dielectric constant by 50–70% resulted in higher apparent surface potential. Moreover, the organic layers (EDA and APTES) in modified flakes with the highest surface potential show higher thicknesses (by 30–40%). Thus, the resulting increase in effective dielectric constant of the tip-flake “capacitor” increases by 40–50% to 1.4 for GO-EDA and 1.5 for MX-APTES flakes as can be estimated from gap thicknesses (70 nm), layer thicknesses, and dielectric constants (Tables S2 and S3).

## Conclusions

By employing advanced probing techniques, we correlated topographical features with variations in chemical composition, surface stiffness, adhesive forces, and surface potential across the pristine and chemically modified flakes, including their edges and defects. The formation of stable Langmuir monolayers of chemically modified flakes, which are prone to agglomeration, and relieving the difficulty of postmodification thin film formation allows for the intricate study of the surface properties of individual flakes and their specific features (inner surface, edges, and defects) rather than bulk materials. Our results indicate that graphene oxide flakes achieve uniform coverage with substantial binding of organic molecules at their edges. In contrast, MXene modified with APTES shows full coverage without excess binding at the edges. Interestingly, dopamine-modified MXene displays nonuniformity, with a decreased concentration near the edges but no excessive binding occurring at the edges or microscopic defects. Among the materials studied, dopamine-modified MXene exhibits the highest surface potential and the lowest stiffness, while dopamine-modified graphene oxide flakes show only a slight increase in surface stiffness.

As observed, functional groups on the surface of graphene oxide appear to be more evenly distributed over the entirety of the flake, allowing for more uniform surface modification. MXene, on the other hand, has an uneven distribution of adsorbed and grafted molecules and therefore can be inferred from the presence of the randomly distributed functional groups on the surface. Comparing both species modified with dopamine via nanoscale characterization illuminates these heterogeneities and differences between flakes. These differences suggest an inherent disparity in the surface reactivity and chemical accessibility between the respective material surfaces.

Chemical modifications significantly enhance the adhesion of MXene flakes, but not for initially adhesive graphene oxide. The surface potential of dopamine-modified MXene demonstrates greater uniformity compared to that of modified graphene oxide and MXene with APTES coverage. Overall, the significant increase in surface potential observed for different organic layers can be related to the increased capacitance due to the increased effective dielectric permittivity within the tip-modified flake capacitors.

In summary, understanding a combination of these nanoscale heterogeneous properties as characterized by diverse scanning probe microscopy modes suggested in this study is essential for advancing our knowledge of how these 2D materials will assemble within complex composite interfaces in polymer nanocomposites and as parts of multicomponent functional devices.

Graphene oxide has a homogeneous arrangement of surface functional groups with preferred adsorption along the edge compared to MXene, which has a heterogeneous surface arrangement across the whole flakes without noticeable edge-preference effects. While surface functionalization of 2D materials is widely explored, nanoscale correlations among topography, mechanical properties, adhesion, and surface potential are difficult to correlate. The use of AFM to correlate interdependent surface properties provides a unique viewpoint into how chemical modifications manifest themselves spatially at the nanoscale down to individual flakes with their features, such as edges, defects, and initial functionality distribution. The formation of ultrathin and uniform versus thicker and variable surface layers with different surface chemistries, grafting density, and dielectric properties on the 2D flakes alters adhesion and electrical properties within stacked heterostructures with deliberate interlayer spacing and sensing abilities. Understanding these localized disparities is key for future interfacial engineering of 2D flakes, and these techniques can be applied to other functionalized molecules and 2D material composites.

## Supplementary Material



## Notes

Experimental data and methods; including XPS binding energy peak assignments; KPFM table; nano-IR calibration details and figures; flake thickness before and after modification and thickness of organic layers; AFM height profiles for corresponding functionalized flake Langmuir monolayers comparing layer thickness; and AFM topographical images and height profiles for single flakes of functionalized MXene with dopamine (PDF)

## References

[1] Zhu Y., Murali S., Cai W., Li X., Suk J. W., Potts J. R., Ruoff R. S. (2010). Graphene and graphene oxide: synthesis,
properties, and applications. Adv. Mater..

[2] Lipatov A., Lu H., Alhabeb M., Anasori B., Gruverman A., Gogotsi Y., Sinitskii A. (2018). Elastic properties of 2D Ti_2_C_2_T_
*x*
_ MXene monolayers and
bilayers. Sci. Adv..

[3] Ren H., Xia X., Sun Y., Zhai Y., Zhang Z., Wu J., Li J., Lui M. (2024). Electrolyte engineering for the mass exfoliation of
graphene oxide across wide oxidation degrees. J. Mater. Chem. A.

[4] Hantanasirisakul K., Gogotsi Y. (2018). Electronic and Optical
Properties of 2D Transition
Metal Carbides and Nitrides (MXenes). Adv. Mater..

[5] Lin Z., Shao H., Xu K., Taberna P.-L., Simon P. (2020). MXenes as
High-Rate Electrodes for Energy Storage. Trends
Chem..

[6] Li X., Huang Z., Shuck C. E., Liang G., Gogotsi Y., Zhi C. (2022). MXene chemistry, electrochemistry and energy storage applications. Nat. Rev. Chem..

[7] Lee A., Shekhirev M., Anayee M., Gogotsi Y. (2024). Multi-year study of
environmental stability of Ti3C2Tx MXene films. Graphene 2D Mater..

[8] Maleski K., Mochalin V. N., Gogotsi Y. (2017). Dispersions of Two-Dimensional
Titanium
Carbide MXene in Organic Solvents. Chem. Mater..

[9] Mozafari M., Soroush M. (2021). Surface functionalization of MXenes. Mater. Adv..

[10] Mannix A. J., Zhang Z. H., Guisinger N. P., Yakobson B. I., Hersam M. C. (2018). Borophene
as a prototype for synthetic 2D materials development. Nat. Nanotechnol..

[11] Hu F., Kim M., Zhang Y., Luan Y., Ho K. M., Shi Y., Wang C. Z., Wang X., Fei Z. (2019). Tailored Plasmons in
Pentacene/Graphene Heterostructures with Interlayer Electron Transfer. Nano Lett..

[12] Kim N. H., Kuila T., Lee J. H. (2013). Simultaneous
reduction, functionalization
and stitching of graphene oxide with ethylenediamine for composites
application. J. Mater. Chem. A.

[13] Ren J., Zhao X., Zhang J., Zhang Q. (2016). Anthraquinone Immobilized
on Reduced Graphene Oxide Sheets with Improved Electrochemical Properties
for Supercapacitors. Int. J. Electrochem. Sci..

[14] Ren H., Kulkarni D. D., Kodiyath R., Xu W., Choi I., Tsukruk V. V. (2014). Competitive Adsorption of Dopamine
and Rhodamine 6G
on the Surface of Graphene Oxide. ACS Appl.
Mater. Interfaces.

[15] Adstedt K., Buxton M. L., Henderson L. C., Hayne D. J., Nepal D., Gogotsi Y., Tsukruk V. V. (2023). 2D Graphene Oxide and MXene Nanosheets
at Carbon Fiber Surfaces. Carbon.

[16] Ma J., Pan J., Yue J., Xu Y., Bao J. (2018). High performance of
poly­(dopamine)-functionalized graphene oxide/poly­(vinyl alcohol) nanocomposites. Appl. Surf. Sci..

[17] Hu X., Qi R., Zhu J., Lu J., Luo Y., Jin J., Jiang P. (2014). Preparation and Properties of Dopamine Reduced Graphene
Oxide and
Its Composites of Epoxy. J. Appl. Polym. Sci..

[18] Heckler J. E., Neher G. R., Mehmood F., Lioi D. B., Pachter R., Vaia R., Kennedy W. J., Nepal D. (2021). Surface Functionalization
of Ti_3_C_2_T_
*x*
_ MXene
Nanosheets with Catechols: Implication for Colloidal Processing. Langmuir.

[19] Xiong R., Kim H. S., Zhang L., Korolovych V. F., Zhang S., Yingling Y. G., Tsukruk V. V. (2018). Wrapping
Nanocellulose
Nets around Graphene Oxide Sheets. Angew. Chem.,
Int. Ed..

[20] Kulkarni D. D., Kim S., Chyasnavichyus M., Hu K., Fedorov A. G., Tsukruk V. V. (2014). Chemical Reduction of Individual Graphene Oxide Sheets
as Revealed by Electrostatic Force Microscopy. J. Am. Chem. Soc..

[21] Chen T. D., Wang J. Q., Wu X. Z., Li Z. P., Yang S. R. (2021). Ethanediamine
induced self-assembly of long-range ordered GO/MXene composite aerogel
and its piezoresistive sensing performances. Appl. Surf. Sci..

[22] Compton O. C., Dikin D. A., Putz K. W., Brinson L. C., Nguyen S. T. (2010). Electrically
Conductive ″Alkylated″ Graphene Paper via Chemical Reduction
of Amine-Functionalized Graphene Oxide Paper. Adv. Mater..

[23] Kwon Y. B., Cho S., Min D. H., Kim Y. K. (2024). Bio-inspired interfacial chemistry
for the fabrication of a robust and functional graphene oxide composite
film. RSC Adv..

[24] Sharma S., Park B. B., Kokkiligadda S., Basak S., Hong S. T., Hur S. H., Chung J. S. (2024). Catechol
grafted rGO/MXene heterosheet
structures for high performance electromagnetic interference shielding
and thermal management applications. Carbon.

[25] Gong Y., Xue P., Wang X., Ma S., Xu X. (2024). Antioxidative ultrafast
light-driven poly­(N-isopropylacrylamide) hydrogel actuator enabled
by (3-aminopropyl)­triethoxysilane-modified MXene and polyvinyl alcohol. J. Mater. Sci..

[26] Ji J. J., Zhao L. F., Shen Y. F., Liu S. Q., Zhang Y. J. (2019). Covalent
stabilization and functionalization of MXene via silylation reactions
with improved surface properties. FlatChem.

[27] Chen C., Xie X. Q., Anasori B., Sarycheva A., Makaryan T., Zhao M. Q., Urbankowski P., Miao L., Jiang J. J., Gogotsi Y. (2018). MoS2-on-MXene Heterostructures
as Highly Reversible Anode Materials for Lithium-Ion Batteries. Angew. Chem. Int. Ed..

[28] Hubbard A. M., Ren Y. X., Papaioannou P., Sarvestani A., Picu C. R., Konkolewicz D., Roy A. K., Varshney V., Nepal D. (2022). Vitrimer Composites:
Understanding the Role of Filler in Vitrimer
Applicability. ACS Appl. Polym. Mater..

[29] Shekhirev M., Shuck C. E., Sarycheva A., Gogotsi Y. (2021). Characterization of
MXenes at every step, from their precursors to single flakes and assembled
films. Prog. Mater. Sci..

[30] Krecker M. C., Bukharina D., Hatter C. B., Gogotsi Y., Tsukruk V. V. (2020). Bioencapsulated
MXene Flakes for Enhanced Stability and Composite Precursors. Adv. Funct Mater..

[31] Parra-Munoz N., Soler M., Rosenkranz A. (2022). Covalent functionalization of MXenes
for tribological purposes-a critical review. Adv. Colloid Interfac.

[32] Gimenez R., Serrano B., San-Miguel V., Cabanelas J. C. (2022). Recent
Advances in MXene/Epoxy Composites: Trends and Prospects. Polymers.

[33] Downes M., Shuck C. E., McBride B., Busa J., Gogotsi Y. (2024). Comprehensive
synthesis of Ti_3_C_2_T_
*x*
_ from MAX phase to MXene. Nat. Protoc..

[34] Hummers W. S., Offeman R. E. (1958). Preparation of Graphitic Oxide. J. Am. Chem. Soc..

[35] Hu K., Gupta M. K., Kulkarni D. D., Tsukruk V. V. (2013). Ultra-Robust Graphene-Oxide
Silk Firoin Nanocomposite Membranes. Adv. Mater..

[36] Flouda P., Shah S. A., Lagoudas D. C., Green M. J., Lutkenhaus J. L. (2019). Highly
Multifunctional Dopamine-Functionalized Reduced Graphene Oxide Supercapacitors. Matter.

[37] Kulkarni D. D., Choi I., Singamaneni S. S., Tsukruk V. V. (2010). Graphene Oxide-Polyelectrolyte
Nanomembranes. ACS Nano.

[38] McConney M. E., Singamaneni S., Tsukruk V. V. (2010). Probing Soft Matter with the Atomic
Force Microscopies: Imaging and Force Spectroscopy. Polym. Rev..

[39] Mohn F., Gross L., Moll N., Meyer G. (2012). Imaging the
charge
distribution within a single molecule. Nat.
Nanotechnol..

[40] Schwartz J. J., Jakob D. S., Centrone A. (2022). A guide to nanoscale IR spectroscopy:
resonance enhanced transduction in contact and tapping mode AFM-IR. Chem. Soc. Rev..

[41] Hussain S. A., Dey B., Bhattacharjee D., Mehta N. (2018). Unique supramolecular
assembly through Langmuir - Blodgett (LB) technique. Heliyon.

[42] Wei L. F., Ma J. Z., Zhang W. B., Pan Z. Y., Ma Z. L., Kang S. L., Fan Q. Q. (2020). Enhanced Antistatic
and Self-Heatable
Wearable Coating with Self-Tiered Structure Caused by Amphiphilic
MXene in Waterborne Polymer. Langmuir.

[43] Fan L., Wen P., Zhao X. W., Zou J. L., Kim F. (2022). Langmuir-Blodgett Assembly
of Ti3C2Tx Nanosheets for Planar Microsupercapacitors. Acs Appl. Nano Mater..

[44] Petukhov D. I., Chumakov A. P., Kan A. S., Lebedev V. A., Eliseev A. A., Konovalov O. V., Eliseev A. A. (2019). Spontaneous MXene monolayer assembly
at the liquid-air interface. Nanoscale.

[45] Flouda P., Inman A., Gumenna M., Bukharina D., Shevchenko V. V., Gogotsi Y., Tsukruk V. V. (2023). Ultrathin
Films
of MXene Nanosheets Decorated by Ionic Branched Nanoparticles with
Enhanced Energy Storage Stability. Acs Appl.
Mater. Inter.

[46] Flouda P., Stryutsky A. V., Buxton M. L., Adstedt K. M., Bukharina D., Shevchenko V. V., Tsukruk V. V. (2022). Reconfiguration of Langmuir Monolayers
of Thermo-Responsive Branched Ionic Polymers with LCST Transition. Langmuir.

[47] Shen J. L., Noh B. I., Chen P. Y., Dai S. Y. (2024). Scanning Probe Nano-Infrared
Imaging and Spectroscopy of Biochemical and Natural Materials. Small Sci..

[48] Chen T. D., Wang J. Q., Wu X. Z., Li Z. P., Yang S. R. (2021). Ethanediamine
induced self-assembly of long-range ordered GO/MXene composite aerogel
and its piezoresistive sensing performances. Appl. Surf. Sci..

[49] Liu G. Z., Liu S., Ma K., Wang H. Y., Wang X. Y., Liu G. P., Jin W. Q. (2020). Polyelectrolyte
Functionalized TiCT MXene Membranes
for Pervaporation Dehydration of Isopropanol/Water Mixtures. Ind. Eng. Chem. Res..

[50] Sangu S. S., Illias N. M., Ong C. C., Gopinath S. C. B., Saheed M. S. M. (2021). MXene-Based
Aptasensor: Characterization and High-Performance Voltammetry Detection
of Deoxynivalenol. Bionanoscience.

[51] Flouda P., Choi J., Buxton M. L., Nepal D., Lin Z. Q., Bunning T. J., Tsukruk V. V. (2023). Synthesis
and assembly of two-dimensional
heterostructured architectures. MRS Commun..

[52] Suk J. W., Piner R. D., An J. H., Ruoff R. S. (2010). Mechanical Properties
of Mono layer Graphene Oxide. ACS Nano.

[53] Zeng G., Dirscherl K., Garnæs J. (2018). Toward Accurate Quantitative Elasticity
Mapping of Rigid Nanomaterials by Atomic Force Microscopy: Effect
of Acquisition Frequency, Loading Force, and Tip Geometry. Nanomaterials.

[54] Chae Y., Kim S. J., Cho S.-Y., Choi J., Maleski K., Lee B.-J., Jung H.-T., Gogotsi Y., Lee Y., Ahn C. W. (2019). An investigation into the factors governing the oxidation
of two-dimensional Ti_3_C_2_T_
*x*
_ MXene. Nanoscale.

[55] Chen J., Wu J. M., Ge H. Y., Zhao D., Liu C., Hong X. F. (2016). Reduced graphene
oxide deposited carbon fiber reinforced
polymer composites for electromagnetic interference shielding. Composites, Part A.

[56] Zeng G. H., Dirscherl K., Garnaes J. (2018). Toward Accurate Quantitative Elasticity
Mapping of Rigid Nanomaterials by Atomic Force Microscopy: Effect
of Acquisition Frequency, Loading Force, and Tip Geometry. Nanomaterials.

[57] Jakob D. S., Li N. X., Zhou H. P., Xu X. J. G. (2021). Integrated Tapping
Mode Kelvin Probe Force Microscopy with Photoinduced Force Microscopy
for Correlative Chemical and Surface Potential Mapping. Small.

[58] Fatkullin M., Cheshev D., Averkiev A., Gorbunova A., Murastov G., Liu J., Postnikov P., Cheng C., Rodriguez R. D., Sheremet E. (2024). Photochemistry dominates
over photothermal effects in the laser-induced reduction of graphene
oxide by visible light. Nat. Commun..

[59] Glatzel T., Gysin U., Meyer E. (2022). Kelvin probe
force microscopy for
material characterization. Microscopy.

[60] Adstedt K., Stojcevski F., Newman B., Hayne D. J., Henderson L. C., Mollenhauer D., Nepal D., Tsukruk V. (2022). Carbon Fiber
Surface
Functional Landscapes: Nanoscale Topography and Property Distribution. ACS Appl. Mater. Interfaces.

[61] Jahng J., Lee S., Hong S. G., Lee C. J., Menabde S. G., Jang M. S., Kim D. H., Son J., Lee E. S. (2023). Characterizing and
controlling infrared phonon anomaly of bilayer graphene in optical-electrical
force nanoscopy. Light Sci. Appl..

[62] Araújo R. L., Neto J. X. L., Henriques J. M., Tromer R. M., Barboza C. A., Oliveira J. I. N., Fulco U. L. (2020). Insights
into solid-state properties
of dopamine and L-Dopa hydrochloride crystals through DFT calculations. Chem. Phys. Lett..

[63] Zhi X., Mao Y. Y., Yu Z. Z., Wen S. P., Li Y., Zhang L. Q., Chan T. W., Liu L. (2015). γ-Aminopropyl
triethoxysilane functionalized graphene oxide for composites with
high dielectric constant and low dielectric loss. Composites, Part A.

