# Supplementary material for: Surface Mapping of Functionalized Two-Dimensional Nanosheets: Graphene Oxide and MXene Materials
Source: Langmuir. 2025 May 12;41(19):11866–81. doi: 10.1021/acs.langmuir.4c05106 (PMC12100715; doi:10.1021/acs.langmuir.4c05106)
Supplement: Supplementary file 1 [file la4c05106_si_001.pdf]

Supplemental Information

Surface Mapping of Functionalized Two-Dimensional Nanosheets: graphene oxide and MXene materials

Madeline L. Buxton [a], Justin Brackenridge [a], Valeriia Poliukhova [a], Dhriti Nepal [b], Timothy J. Bunning [b], Vladimir V. Tsukruk [a *]

*vladimir@mse.gatech.edu

[a] School of Materials Science and Engineering, Georgia Institute of Technology, Atlanta, GA, 30332, United States

[b] Air Force Research Lab, Materials and Manufacturing Directorate, 2941 Hobson Way, WPAFB, Ohio, 45433, United States

Table S1: XPS binding energy peak assignments[1,2,3,4]

Binding Energy (eV)	Peak Assignment
288.5	C=O/C=OO
286.5	C-O
285.5	C-N
284.5	C-C/C=C
283	C-Si
283	C-Ti-O
282	C-Ti

Table S2: KPFM average surface potentials and dieelectric constants

Sample	Average surface potential (mV)	Dielectric constant
GO	98.7 ± 0.21	-
GO DOPA	142.2 ± 0.11	9
GO EDA	202.8 ± 0.33	15
MX	151.9 ± 0.44	-
MX DOPA	172.1 ± 0.09	9
MX APTES	199.4 ± 0.32	13

Table S3: Flake thickness before and after modification and thickness of organic layers

**Sample**	Thickness of flakes (nm)	Thickness of organic layers (nm)
GO	1.3 ± 0.2	NA
GO DOPA	4.9 ± 1.4	1.8 ± 0.7
GO EDA	6.1 ± 1.6	2.4 ± 0.8
MX	3.6 ± 0.5	NA
MX DOPA	7.8 ± 3.1	2.1 ± 1.5
MX APTES	8.9 ± 2.5	2.7 ± 1.2

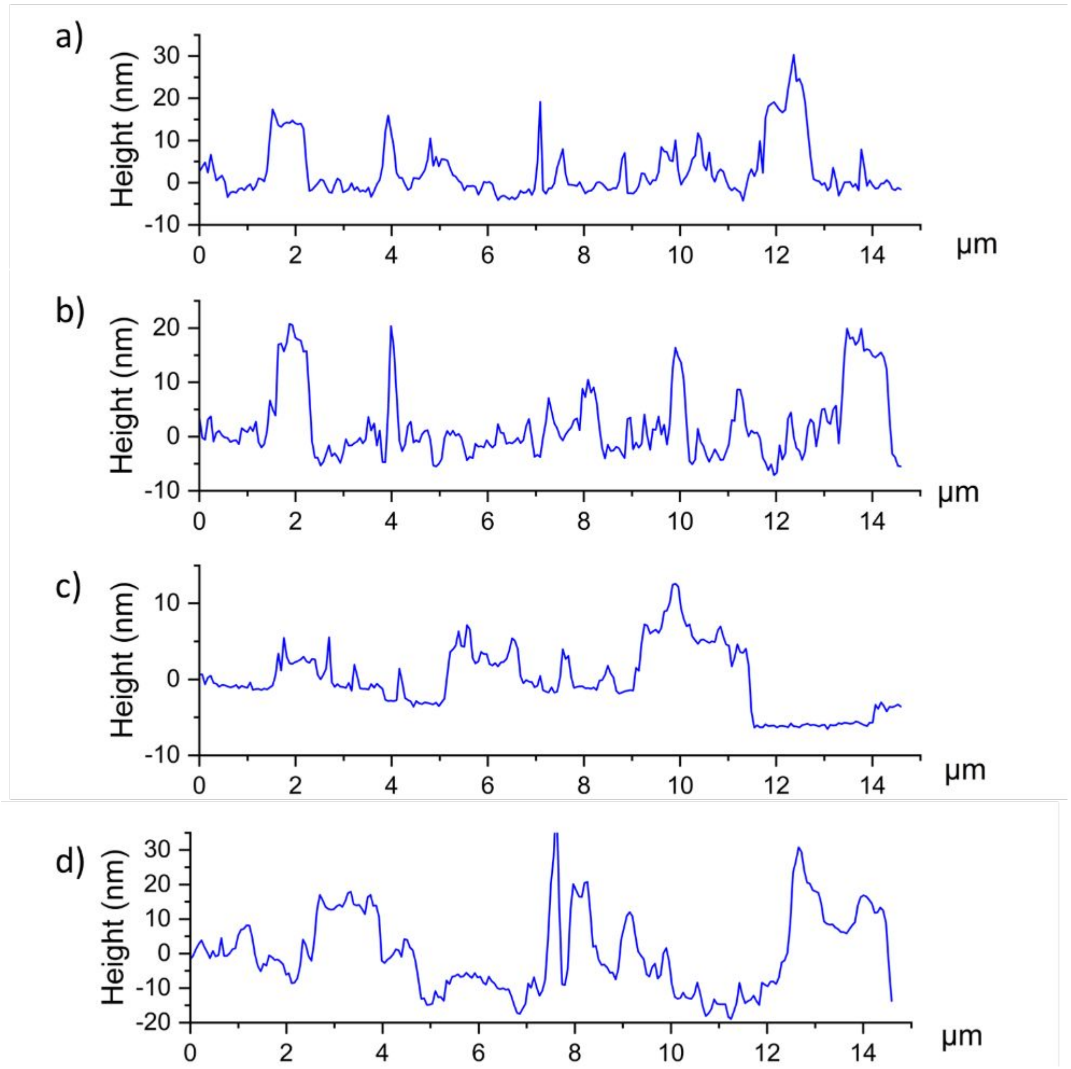

Figure S1: AFM height profiles for corresponding functionalized flake Langmuir monolayers comparing layer thickness. Flake profiles for films of graphene oxide with EDA (a), graphene oxide with dopamine (b), MXene with dopamine (c) and MXene with APTES (d).

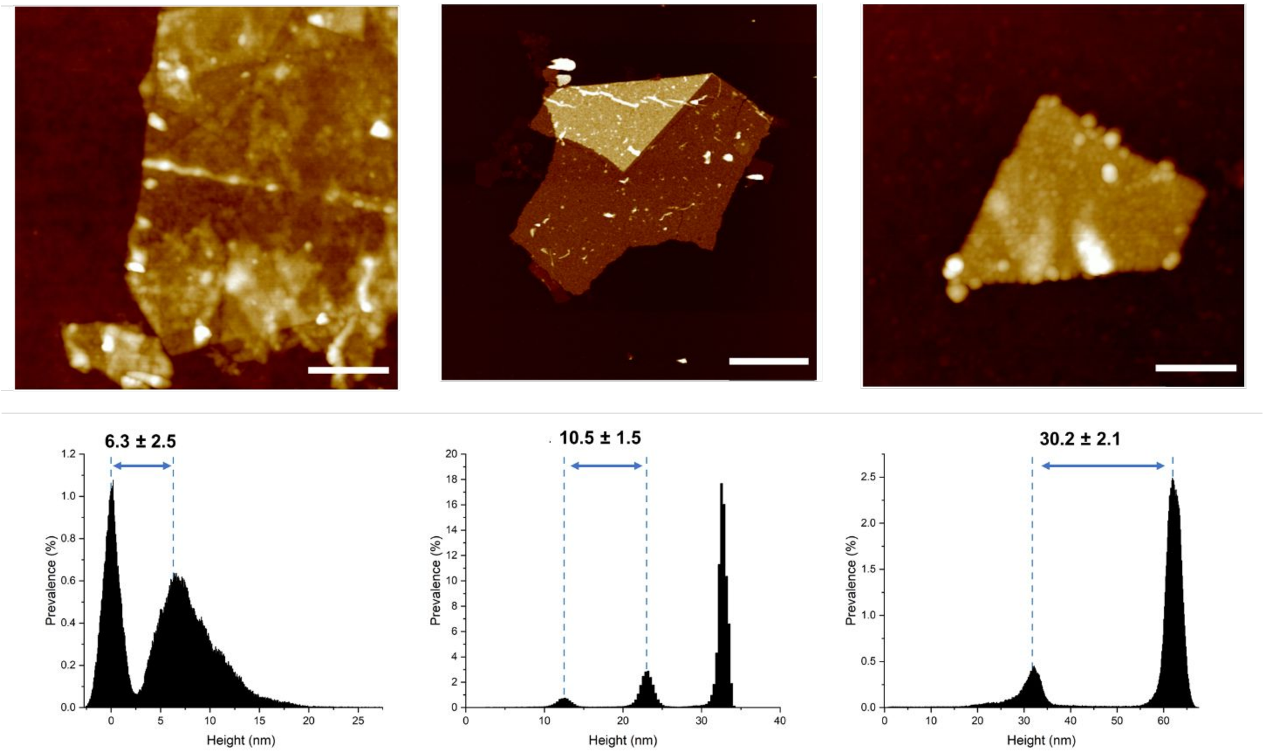

Figure S2: AFM topographical images and height profiles for single flakes of functionalized MXene with Dopamine. The concentration ratio of dopamine to MXene is changed from left to right, 0.5:1 mg/ml, 1:1 mg/ml, and 2:1 mg/ml. Corresponding height histograms of single layers, as determined by topography. The lateral scale bar for images is 330 nm, 2.0 µm, and 330 nm respectively.

An additional peak around 33 nm can be seen in the histogram of the sample at a concentration of 1:1 mg/ml dopamine to MXene (central image and diagram) due to the bilayer formed as a resulting on the presence of additional overlapped flake.

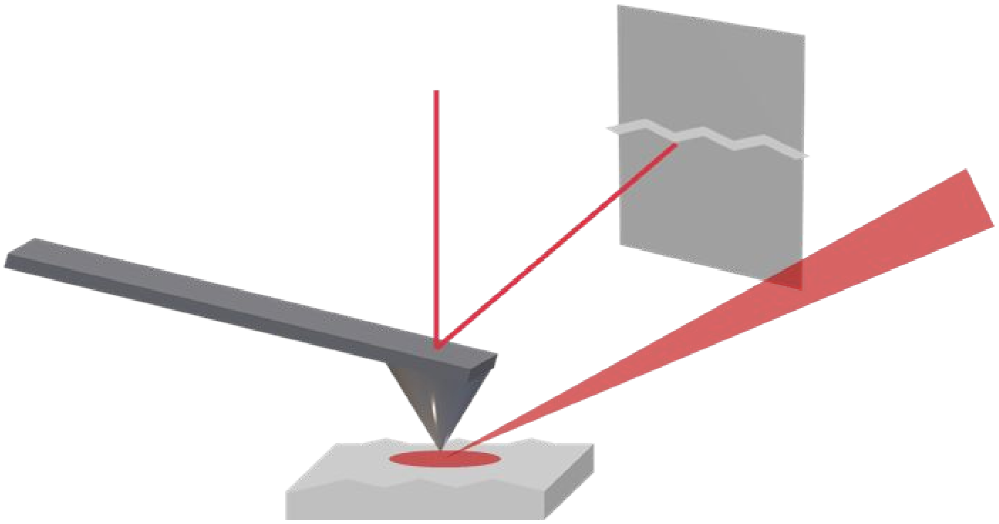

Figure S3: Nano-IR schematic showing AFM cantilever and IR laser irradiation.

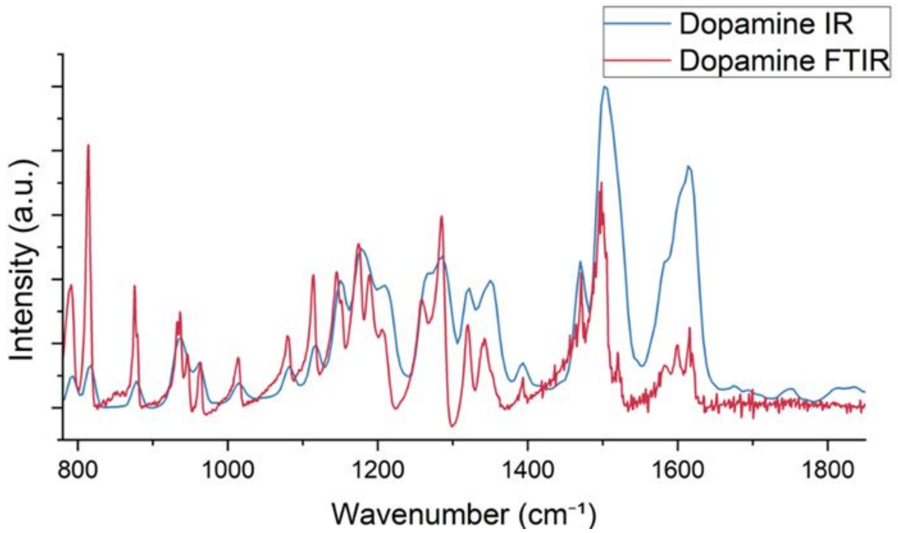

Figure S4: Comparison of Nano-IR spectrum and FTIR spectrum of drop cast dopamine film.

Calibration of Nano-IR data

Nano-IR measurements are the result of localized thermal expansion in the range of 1nm to 10s of picometers and heat diffuses from the sample between laser pulses.[5] The laser intensity and scan location choice were made to ensure repeatability and accurate measurement while maintaining sufficient signal-to-noise ratio supported by calibration.

To verify that the Nano-IR signal corresponds to accurate peak analysis, calibration was done on a known sample with a well-established IR signature. Organic molecules have a stronger signal in the fingerprint region that is within the nano-IR range and thus provide clarity on the quality and accuracy of the Nano-IR measurements. On the same drop cast dopamine sample both the FTIR and the Nano-IR measurements are compared. We can see that the peaks of both measurements follow the same pattern. There tends to be a slight broadening of the nano-IR peaks as well as a slight blue shift at values greater than 1500 cm-1, indicating the influence of surface grafting. Intensity ratios are altered with the strongest Nano-IR signal above 1400 cm^{-1}. Overall, excellent overlap indicates reputable and valid nano-IR data.

Between each sample, the Nano-IR laser needs to be realigned and calibrated. Therefore, instead of looking at absolute intensities, comparing peak positions of each respective graph is critical. For each 2D material, two samples were made of different thicknesses. The thin sample is made via LB deposition and coated onto a silicon wafer, and the thick sample is drop cast; therefore, thickness ranges are 10-30 nm and 200-300 nm, respectively. This way substrate interference is negligible on the thick samples greater than the penetration depth of the IR laser. While minimizing as such the peaks can be attributed to the materials and therefore compared to thin samples to confirm and minimize substrate interference. However, at the consequence of no flake-to-flake distinction or order. The thick samples confirm the presence of peaks with a better signal-to-noise ratio that improves the analysis of the thin samples.

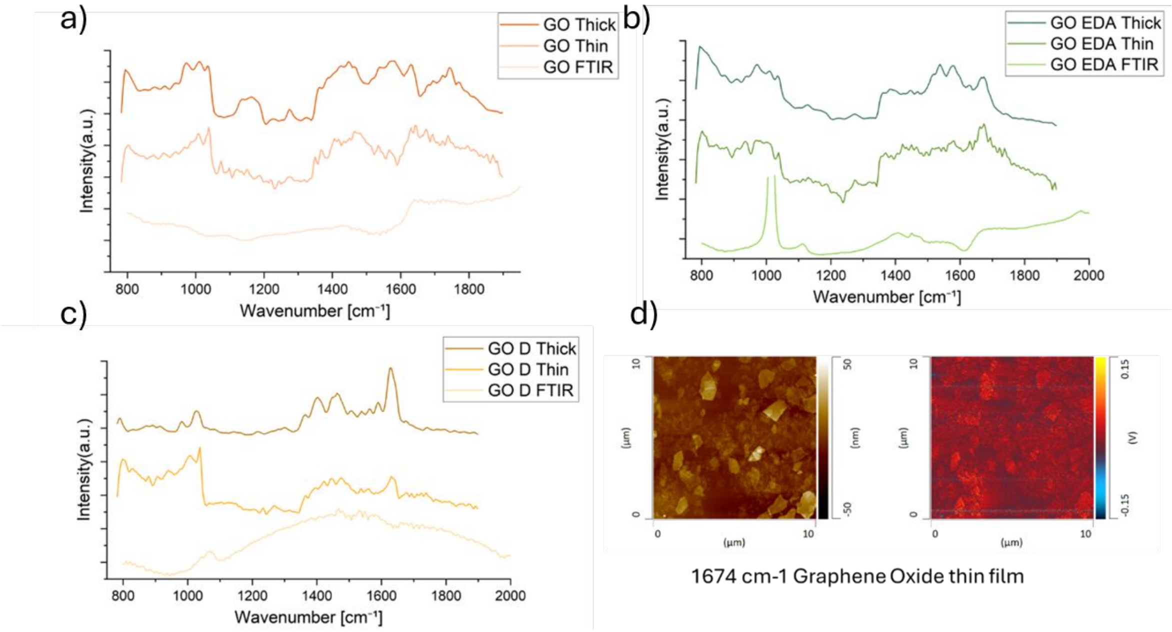

Figure S5: Nano-IR spectra comparison of graphene oxide with traditional FTIR data. For each graph data is included for Nano-IR of Langmuir monolayer film, Nano-IR of drop cast (thick) film, and ATR-FTIR spectra: a) unmodified graphene oxide, b) graphene oxide with EDA, c) graphene oxide with dopamine, and d) Nano-IR mapping of graphene oxide thin film at 1674 cm^{-1}, lateral scale bar is 10 um.

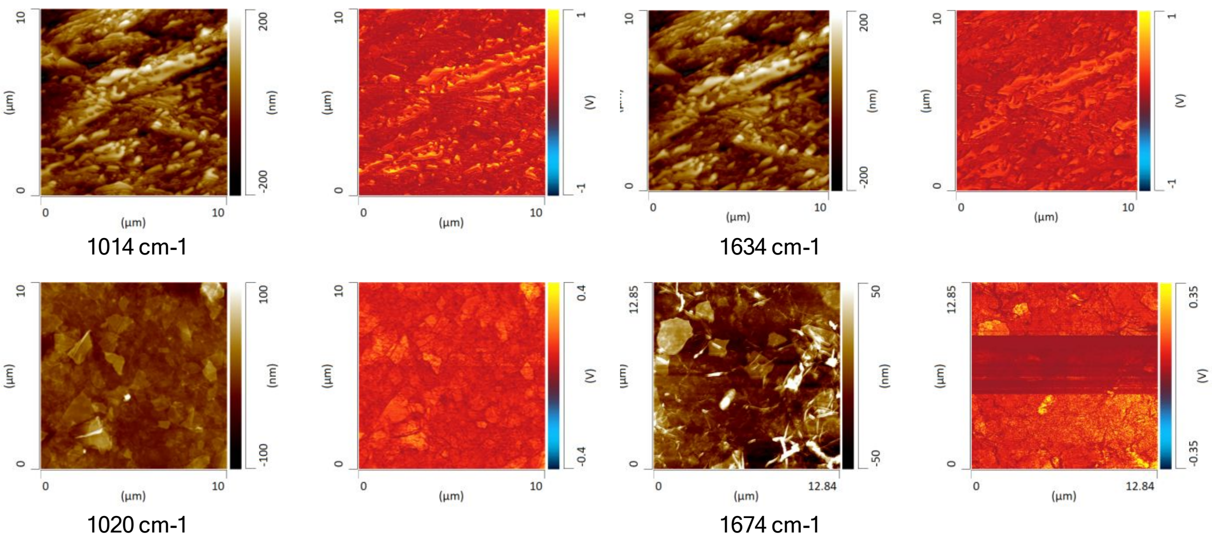

Figure S6: Nano-IR mapping of graphene oxide modified by dopamine. Thick drop cast films (top) and thin Langmuir monolayers (bottom) at various wavenumbers noted underneath.

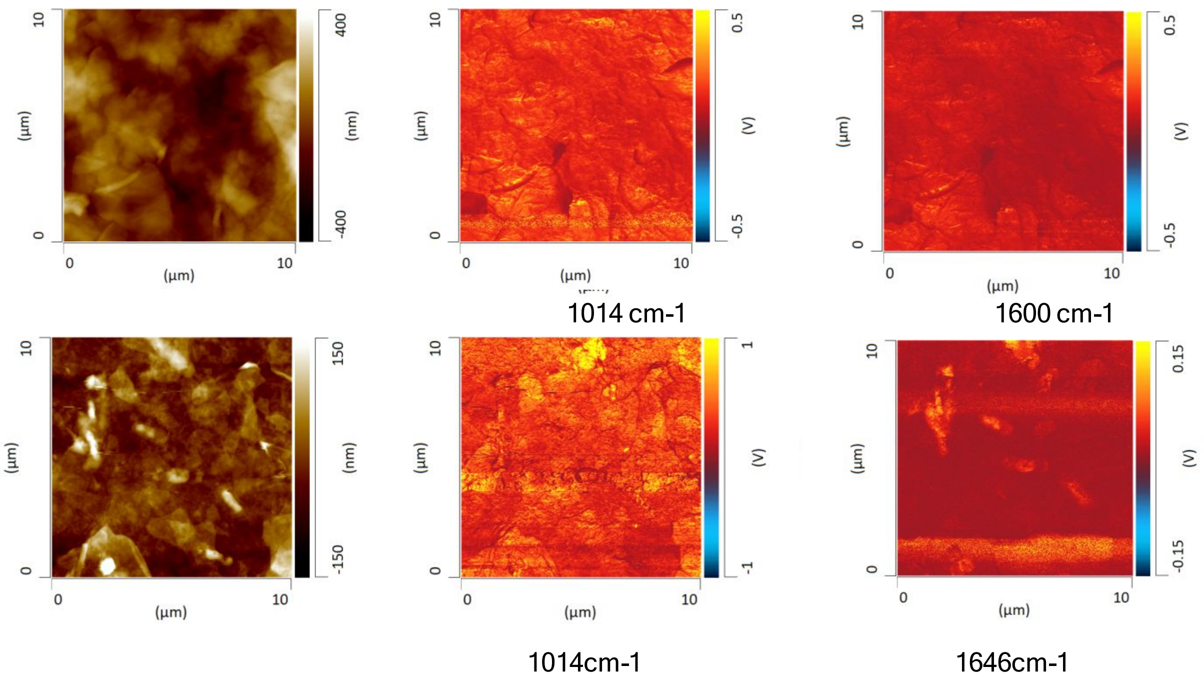

Figure S7: Nano-IR mapping of graphene oxide modified by EDA. Thick drop cast films (top) and thin Langmuir monolayers (bottom) at various wavenumbers are noted underneath.

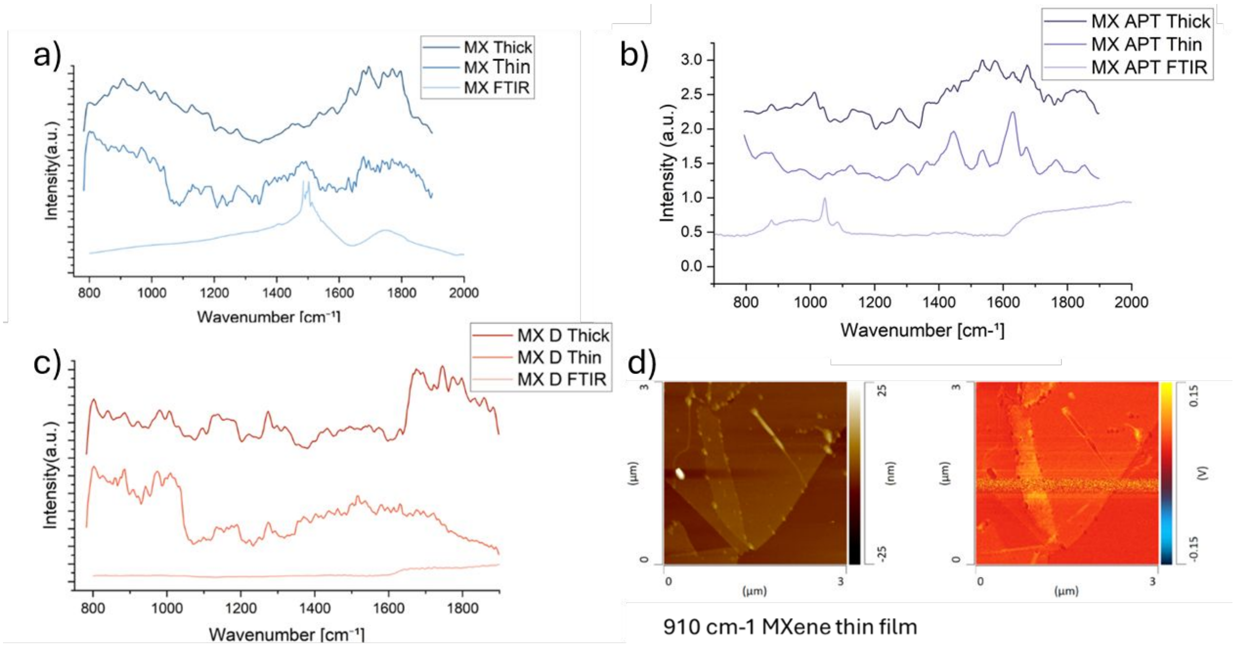

Figure S8: Nano-IR spectral comparison of MXene versus FTIR bulk measurements. For each graph data is included for Nano-IR of Langmuir monolayer (thin) film, Nano-IR of dropcast (thick) film, and ATR-FTIR spectra: a) unmodified MXene, b) MXene with APTES, c) MXene with dopamine, and d) Nano-IR mapping of MXene thin film at 910 cm^{-1}, scale bar is 3 um.

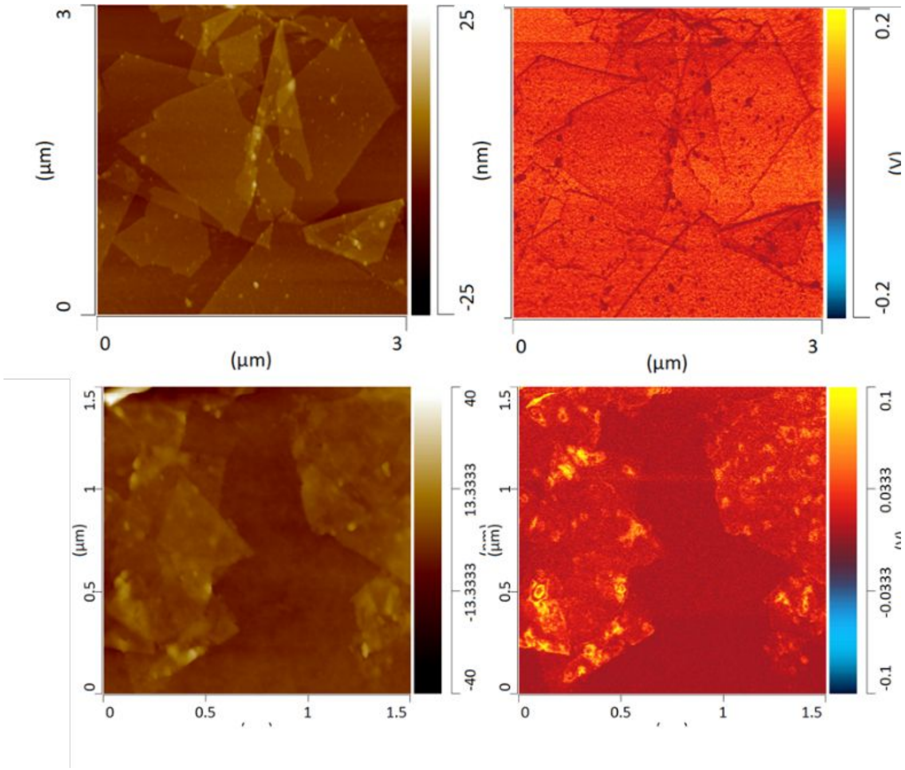

Figure S9: Nano-IR mapping of MXene Langmuir monolayers at 1600 cm^{-1}. Unmodified MXene (top) and MXene with dopamine functionalized at a pH of 10.5 (bottom).

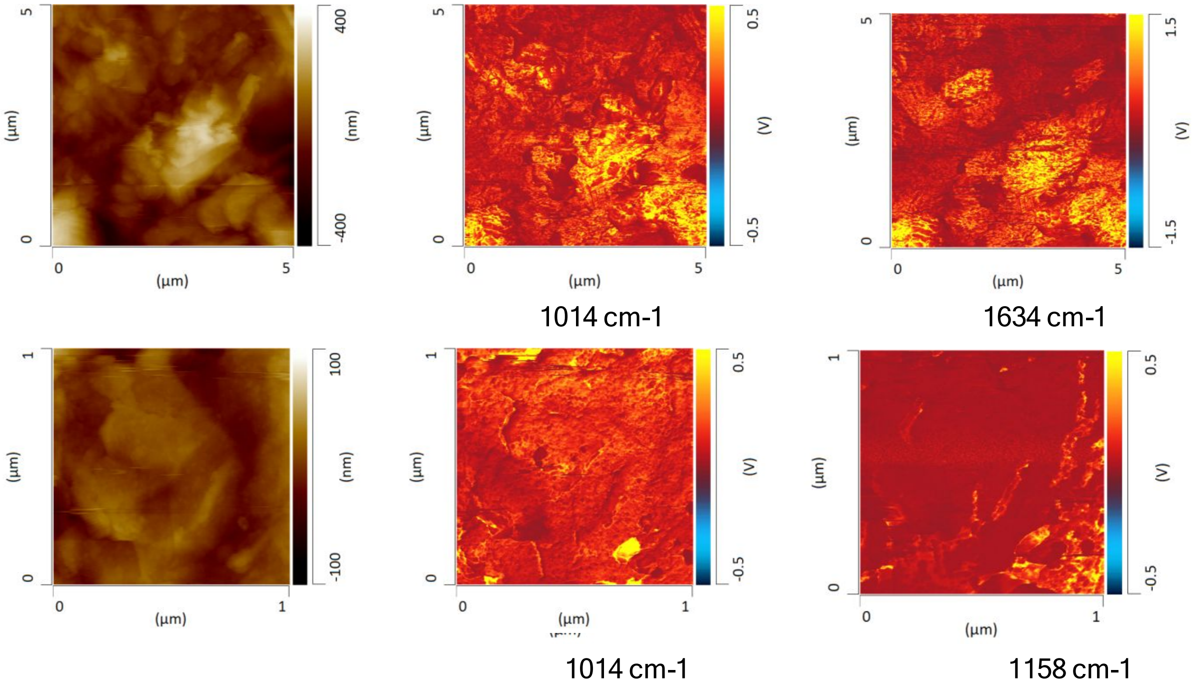

Figure S10: Nano-IR mapping of MXene modified by dopamine. Thick drop cast films (top) and thin Langmuir monolayers (bottom) at various wavenumbers are noted underneath.

References

[1] Rodenstein, M.; Zürcher, S.; Tosatti, S. G. P.; Spencer, N. D. Fabricating Chemical Gradients on Oxide Surfaces by Means of Fluorinated, Catechol-Based, Self-Assembled Monolayers. *Langmuir* **2010**, *26* (21).

[2] Riazi, H.; Anayee, M.; Hantanasirisakul, K.; Shamsabadi, A. A.; Anasori, B.; Gogotsi, Y.; Soroush, M. Surface Modification of a MXene by an Aminosilane Coupling Agent. *Adv Mater Interfaces* **2020**, *7* (6).

[3] Lee, G. S.; Yun, T.; Kim, H.; Kim, I. H.; Choi, J.; Lee, S. H.; Lee, H. J.; Hwang, H. S.; Kim, J. G.; Kim, D. W.; et al. Mussel Inspired Highly Aligned $Ti_3C_2T_x$ MXene Film with Synergistic Enhancement of Mechanical Strength and Ambient Stability. *Acs Nano* **2020**, *14* (9), 11722-11732.

[4] Janica, I.; Montes-García, V.; Urban, F.; Hashemi, P.; Nia, A. S.; Feng, X. L.; Samorì, P.; Ciesielski, A. Covalently Functionalized MXenes for Highly Sensitive Humidity Sensors. *Small Methods* **2023**, *7* (8), e2201651.

[5] Schwartz, J. J.; Jakob, D. S.; Centrone, A. A guide to nanoscale IR spectroscopy: resonance enhanced transduction in contact and tapping mode AFM-IR. *Chem Soc Rev* **2022**, *51* (13), 5248-5267.
